# One size does not fit all: Customizing MCMC methods for hierarchical models using NIMBLE

**DOI:** 10.1002/ece3.6053

**Published:** 2020-02-14

**Authors:** Lauren C. Ponisio, Perry de Valpine, Nicholas Michaud, Daniel Turek

**Affiliations:** ^1^ Department of Entomology University of California Riverside CA USA; ^2^ Department of Environmental Science, Policy, and Management University of California Berkeley CA USA; ^3^ Department of Mathematics and Statistics Williams College Williamstown MA USA

**Keywords:** dynamic occupancy, latent states, Markov chain Monte Carlo, multispecies occupancy, N‐mixture

## Abstract

Improved efficiency of Markov chain Monte Carlo facilitates all aspects of statistical analysis with Bayesian hierarchical models. Identifying strategies to improve MCMC performance is becoming increasingly crucial as the complexity of models, and the run times to fit them, increases. We evaluate different strategies for improving MCMC efficiency using the open‐source software NIMBLE (R package nimble) using common ecological models of species occurrence and abundance as examples. We ask how MCMC efficiency depends on model formulation, model size, data, and sampling strategy. For multiseason and/or multispecies occupancy models and for N‐mixture models, we compare the efficiency of sampling discrete latent states vs. integrating over them, including more vs. fewer hierarchical model components, and univariate vs. block‐sampling methods. We include the common MCMC tool JAGS in comparisons. For simple models, there is little practical difference between computational approaches. As model complexity increases, there are strong interactions between model formulation and sampling strategy on MCMC efficiency. There is no one‐size‐fits‐all best strategy, but rather problem‐specific best strategies related to model structure and type. In all but the simplest cases, NIMBLE's default or customized performance achieves much higher efficiency than JAGS. In the two most complex examples, NIMBLE was 10–12 times more efficient than JAGS. We find NIMBLE is a valuable tool for many ecologists utilizing Bayesian inference, particularly for complex models where JAGS is prohibitively slow. Our results highlight the need for more guidelines and customizable approaches to fit hierarchical models to ensure practitioners can make the most of occupancy and other hierarchical models. By implementing model‐generic MCMC procedures in open‐source software, including the NIMBLE extensions for integrating over latent states (implemented in the R package nimbleEcology), we have made progress toward this aim.

## Graphical abstract

Improved efficiency of Markov chain Monte Carlo facilitates all aspects of statistical analysis with Bayesian hierarchical models. We identify strategies to improve MCMC performance, focusing on common ecological models of species occurrence and abundance using open‐source software NIMBLE and JAGS.

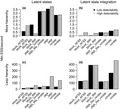

## 1 INTRODUCTION

Application of hierarchical statistical models for analyzing complex ecological data has grown rapidly over roughly the last twenty years (Hobbs & Hooten, 2015; Kéry & Royle, 2016; Royle & Dorazio, 2008). Fundamentally, hierarchical models allow one to account for nonindependence among data by describing a hierarchy of relationships between observations, underlying ecological patterns or processes, and parameters which govern those patterns or processes (Cressie, Calder, Clark, Hoef, & Wikle, 2009). Examples include state‐space time‐series models, spatial models, capture–recapture models, occupancy models, and abundance models (Dorazio & Royle, 2005; Dorazio, Royle, Soderstrom, & Glimskar, 2006; Kéry & Royle, 2016; MacKenzie, Bailey, & Nichols, 2004; MacKenzie et al., 2002, 2006; Rivot, Prévost, Parent, & Bagliniere, 2004; Royle, 2004; Royle & Young, 2008).

Estimation and inference for hierarchical models, however, are not simple. A widely used method is Markov chain Monte Carlo (MCMC) in a Bayesian framework (Brooks, Gelman, Jones, & Meng, 2011; Ellison, 2004). Alternatives to MCMC include Laplace approximation (e.g., TMB Kristensen, Nielsen, Berg, Skaug, & Bell, 2016) and integrated nested Laplace approximation (e.g., INLA, Rue, Martino, & Chopin, 2009; Rue et al., 2017), but here, we focus on MCMC as a widely used, customizable approach. MCMC algorithms sample from the posterior distribution of parameters and latent (unknown) ecological states given the observed data and assumptions about the prior distribution of parameters. More simply, they explore the range of conditions that might explain the data. A major limitation of MCMC is that when a model has hundreds or thousands of latent states and parameters, which may be highly correlated in the posterior distribution, MCMC can require hours, days, or weeks to run. This limits research efficiency, but more importantly, it limits research quality by constraining the range of models that can be compared and the potential for using simulations to check estimation performance, cross‐validation, or other layers of computational analysis (Hooten & Hobbs, 2015).

MCMC is not a single algorithm but rather a large family of algorithms that can be combined flexibly. Statistical researchers have elaborated many MCMC sampling strategies for many kinds of models, and they have pursued theoretical results on MCMC mixing—how well the posterior distribution is explored—and how MCMC mixing scales with the size of a model or data (Gilks & Roberts, 1995; Yu & Meng, 2011). Though these theoretical results are typically limited to simple models and lack consideration of computational costs, these studies suggest that there is no universally best strategy (Gilks & Roberts, 1995; Turek, Valpine, Paciorek, & Anderson‐Bergman, 2017; Yu & Meng, 2011). Instead, the success of customizing sampling strategies for particular models suggests that the best strategies may be problem‐specific (Turek et al., 2017).

The recognition that different sampling strategies may work well for different models points to commonly used software tools as a hindrance to efficient MCMC. Tools such as WinBUGS and OpenBUGS (collectively “BUGS”) and JAGS have revolutionized statistical practice in ecology and other fields by putting MCMC in the hands of nonspecialists, in part because the BUGS syntax is relatively easy to read and adapt (Lunn, Jackson, Best, Spiegelhalter, & Thomas, 2012; Plummer, 2015, 2003; Surhone, Tennoe, & Henssonow, 2010). Other software packages that do not use the BUGS language include Stan, which implements Hamiltonian Monte Carlo methods (HMC; Betancourt & Girolami, 2013; Monnahan, Thorson, & Branch, 2017), as well as numerous other packages that provide sampling strategies for general models or specialized strategies for narrower models, among which we note PyMC (Salvatier, Wiecki, & Fonnesbeck, 2016), MCMCpack (Martin, Quinn, & Park, 2011), spBayes (Finley, Banerjee, & Carlin, 2007), and MCMCglmm (Hadfield, 2010). However, these packages generally prescribe the MCMC methods to be used or offer a small range of choices for expert users. A comparatively new package, NIMBLE (“Numerical Inference for hierarchical Models using Bayesian and Likelihood Estimation,” de Valpine et al., 2017), adopts nearly the same model language as BUGS and JAGS but makes it extensible and supports customization of sampling methods. Provided as R package nimble (NIMBLE Development Team, 2019), it provides a workflow in R with code generation of C++ for efficiency.

Beyond limiting the MCMC sampling strategies applied to a model, hierarchical modeling software often limits the way models can be written, which is important because different ways to write the same model can yield different MCMC performance. A simple example is centered and noncentered parameterizations (Papaspiliopoulos, Roberts, & Skld, 2007). A more complicated example occurs when one wants to analytically marginalize some latent states out of the model by direct summation or numerical integration while using MCMC to sample others. Summing over the latent states in a hidden Markov model for multistate or multi‐event capture–recapture can yield orders‐of‐magnitude improvement in computational efficiency (Turek, Valpine, & Paciorek, 2016). Whereas BUGS and JAGS use a closed model language, NIMBLE supports extensibility of models, making such customizations possible.

In this study, we ask how different strategies for MCMC sampling, different kinds of model structures, and alternative ways to formulate equivalent models all impact MCMC efficiency for common models in ecology and evolution. We test whether efficiency is increased by (a) simplifying model structure, (b) block sampling (e.g., joint sampling of parameters), (c) different types of samplers, and (d) summing over latent states. Based on typical results from the statistical literature, we expect the MCMC efficiency of different strategies will be model‐specific (Browne, Steele, Golalizadeh, & Green, 2009; Solonen et al., 2012), so we examine the interaction of these strategies with different models, focusing on occupancy and N‐mixture models (MacKenzie et al., 2006; Royle & Kéry, 2007; Royle, 2004).

Just over a decade after occupancy models were introduced, they are being used to model species ranging from bees (M'Gonigle, Ponisio, Cutler, & Kremen, 2015) to tigers (Hines et al., 2010) with a great variety of model complexity (Bailey, MacKenzie, & Nichols, 2014; Denes, Silveira, & Beissinger, 2015). Estimating abundance and site occupancy is a critical challenge for most subdisciplines in ecology and evolution concerned with quantifying population dynamics including metapopulation, endangered species, and invasion biology. However, occupancy and N‐mixture models can lead to high‐dimensional MCMC algorithms that can mix slowly, requiring lengthy run times. Standard hierarchical modeling implementations of these models include latent states for the true occupancy state or number of individuals at each site in each closed season, as well as random effects at the level of species, sites, and/or observations. Together, these can yield hundreds or thousands of dimensions that require MCMC sampling.

To examine how to increase model estimation efficiency, we focus on software using the BUGS language including NIMBLE (NIMBLE Development Team, 2019) as well as JAGS (Plummer, 2015). Within NIMBLE, models can be extended with new functions and distributions, which provides enormous flexibility in how models are written. In addition, MCMC can be extended with new sampler configurations and entirely new samplers. Though it is out of the scope of this study to compare all available MCMC software, we focus on NIMBLE because it allows us to examine the efficiency of MCMC customizations in which we are interested, and JAGS to allow comparison to this widely used tool that uses nearly the same model language.

## 2 MATERIALS AND METHODS

We focus on four models—three occupancy and one N‐mixture, (Table 1, Appendix 1)—that are commonly employed in ecology and evolution. The efficiency of sampling strategies may depend on model structure, model size, and the data. To explore the effect of model structure, for each model we created a version with and without some component of hierarchical structure and the associated hyperparameters. To examine the effect of different ways to write the same model, for each case we created a model where we sampled latent states and an equivalent model where we integrate out the latent states to limit MCMC sampling to top‐level parameters (Turek et al., 2016). To explore the effect of different sampling strategies, we created a variety of sampler configurations in NIMBLE, including some that use block sampling as well as NIMBLE's default samplers and a sampler configuration similar to that of JAGS. In one occupancy model, we simulated the data and were, therefore, able to include a scenario with high and low detectability of individuals to explore the effect of changing the parameter values and data on the efficiency of samplers. We next provide more details on each of these contrasts, after which we describe how we compare performance among MCMC methods.

**Table 1:** Summary of the occupancy and N‐mixture model case studies used to explore MCMC efficiency

Model	Description	Data	Top‐level parameters	Latent states	Blocking	Reference
Occupancy: Single‐species, multiseason (Eqs. (A1), (A2))	Colonization and persistence of a single‐species across years	Simulated: 15 years of data across 100 sites, each sampled 5 times. Simulated with high (p = 0.73) and low (p = 0.27) detectability. 7,500 possible detections.	+H: 7 −H: 4	LS sampled (low p): 1,069 LS sampled (high p): 965 LS integrated (low and high p): 0	Block persistence (ϕ) and colonization (γ) parameters for each year	Modified from (Kery & Schaub, 2012) chpt 13.5.1
Occupancy: multispecies, single‐season (Eqs. (A3), (A4), (A5))	Occupancy model of multiple bird species examining the effect of wildlife management and habitat characteristics	1 year of data across 70 sites, each sampled 3–4 times for 58 species. 12,644 possible detections.	+H: 20 −H: 10	LS sampled: 2,964 LS integrated: 0	Block species‐specific slopes and intercepts	Ponisio et al. (2019)
Occupancy: multispecies, multiseason (Eqs. (A6), (A7), (A8), (A9), (A10), (A11))	Colonization and persistence of multiple bee species examining the effect of local and landscape variables on population dynamics	10 years of data across 31 sites, each sampled 2–7 times for 49 species. 30,527 possible detections.	+H: 38 −H: 27	LS sampled: 14,264 LS integrated: 0	Block species‐specific slopes and intercepts	(Kery & Schaub, 2012), chpt 6.11.1
N‐mixture: Zero‐inflated (Eqs (A12), (A13), (A14))	Zero‐inflated N‐mixture model of the abundance of great tits using breeding bird surveys across Switzerland	Great tit counts across 267 1‐km^2^ quadrats on a grid, surveyed 2–3 times a year in 2013. Grid covers a little over 41,000 km^2^. 789 possible detections	+H: 28 −H: 25	LS sampled: 263 LS integrated: 0	Block intercept and slopes	(Kéry & Royle, 2016), chpt 6.11.1

Note

For the columns for top‐level parameters and latent states, we break down each example by the different combinations of model structures and sampling strategies: (a) more hierarchical (+H), (b) less hierarchical (−H), (c) latent states (LS) sampled, and (d) LS integrated.

### 2.1 Model choices

We focus on three occupancy models including a single‐species, multiseason; multispecies, single‐season; and multispecies, multiseason (Table 1, see Appendix 1 for each model's full details). The two multiseason examples use dynamic occupancy models (Royle & Kéry, 2007). The single‐species, multiseason example (modified from Kery & Schaub, 2012) uses simulated data (Table 1, Appendix 1.1). The multispecies, single‐season occupancy example is from Zipkin, Royle, Dawson, and Bates (2010), a study surveying a bird community in Catoctin Mountain Park (CATO) and Frederick City Watershed Cooperative Wildlife Management Area (FCW) with a variety of explanatory variables for habitat suitability (Table 1, Appendix 1.2). The multispecies multiseason example is from Ponisio, Valpine, and MGonigle, L.K. & Kremen, C. (2019), a study surveying a bee community across restored habitat in Northern California, USA, and a large number of explanatory variables and their interactions on detection, colonization, and persistence (Table 1, Appendix 1.3). Finally, for an example N‐mixture model, we followed the example of Kéry and Royle (2016) modeling the abundance of great tits from breeding bird survey data across Switzerland (Table 1, Appendix 1.4). Abundance is modeled as a zero‐inflated Poisson (ZIP), where the zero inflation accounts for unsuitable sites (structural zeros). Latent abundance depends on elevation and habitat‐related explanatory variables, and detection probability depends on site‐ and survey‐related characteristics (including some interactions). Variants of each model are described below.

### 2.2 Model structure

For each model, we identified model terms for which an analyst might assume either there is or is not unexplained heterogeneity in parameters. Without heterogeneity, a single parameter is sufficient. With heterogeneity, different parameters for different parts of the data are assumed to follow a shared distribution, typically with hyperparameters, yielding an additional hierarchical layer in the model. Incorporating multiple sources of variation in this way is a common practice in Bayesian hierarchical modeling and indeed a primary motivation for it. However, it is also common to see pragmatic assumptions of where unexplained heterogeneity will not be modeled. Additional hierarchical structure has major implications for the difficulty of MCMC sampling. For these reasons, we compared MCMC performance for more hierarchical and less hierarchical versions of each model.

For each model, the component with more or less hierarchy corresponded to species‐, year‐, site‐, or survey‐specific parameters, along with the hyperparameters for their common distributions. For the single‐species, multiseason occupancy model, we changed whether there is hierarchical structure for year‐specific persistence and colonization probabilities (Table 1, Appendix 1.1, Eqs. (A1), (A2)). For the multispecies, single‐season occupancy model, we changed whether there are common distributions for species‐specific coefficients for the effect of habitat characteristics on occupancy and detection (Table 1, Appendix 1.2, Eqs. (A3), (A4) v. Eq. A5). For the multispecies, multiseason occupancy model, we also changed whether there is hierarchical structure in the species‐specific coefficients drawn from common distributions for the effects of habitat proximity and quality on persistence and colonization (Table 1, Appendix 1.3, Eqs. (A7), (A8), (A9) v. Eq. A10). Lastly, in the N‐mixture model, we changed whether there is hierarchical structure in site and survey‐specific coefficients on both abundance and detection [Table 1, Appendix 1.4, Eqs. (A11), (A12), Kéry & Royle, 2016). The models including species‐, year‐, site‐, or survey‐specific parameters drawn from common distributions are “more hierarchical” in comparison with the models excluding those terms. Our *a priori* expectation is that sampling models with more hierarchical models will always be less efficient than their less hierarchical counterparts.

### 2.3 Model size

For each model, we wrote custom distributions in NIMBLE to directly sum probabilities over discrete latent states, that is, to marginalize over them. However, the implications of this marginalization differed for each model. For the single‐species, multiseason model, we use a hidden Markov model probability summation across the discrete latent state (occupied vs. unoccupied) across all times for a given site. Hidden Markov models are a general class of models for noisy data of system states that change stochastically, and they encompass many ecological models (Gimenez et al., 2007; Zucchini, MacDonald, & Langrock, 2017). Hidden Markov models are the same as state‐space models but historically have been used for discrete‐state systems. For the multispecies, single‐season model, we simply sum over the two possible occupancy states for each species at each site, much like a zero‐inflated model. For the multispecies, multiseason model, we sum the latent states through time for a species at a site. For the N‐mixture model, we sum over the range of values of *N*, the true local abundance at each site, using Meehan, Michel, and Rue (2017)’s recursive algorithm. Meehan et al. (2017) showed that using R‐INLA, this recursive algorithm was more numerically stable and efficient for estimating N‐mixture models. Still, the N‐mixture case is the most computationally demanding summation because it may need to cover a large range of *N* values. The range of relevant values extended from the lowest 0.00001 quantile to the highest 0.99999 quantile of *N* given an observed count, across all counts. This range is heuristic but should include effectively all relevant probability in the summation. Extensions for latent state integration are now available in R package nimbleEcology (Goldstein, Turek, Ponisio, & Valpine, 2019).

Our *a priori* expectation was that integrating over latent states would increase efficiency in some cases but not in others. It is well known in MCMC theory and practice that sometimes it is helpful to deliberately introduce auxiliary variables, even if they can be analytically integrated over, while in other cases the opposite is true. MCMC sampling can be viewed as a form of Monte Carlo integration. Hence, directly integrating vs. sampling represent two ways to numerically handle a dimension of a hierarchical model, and one or the other may be more efficient depending on the context.

### 2.4 Data

With the single‐species, multiseason example (Table 1, Appendix 1.1), we were able to modify the data because it is simulated. We simulated the data with high (*p* = .73) and low (*p* = .27) detectability. We expect that because a lower detection probability will result in more nondetections, the latent states for more site‐years will need to be sampled (Table 1), thereby decreasing efficiency.

### 2.5 MCMC sampling strategies

We fit each model using a variety of MCMC sampling strategies. Before summarizing these strategies, we briefly introduce the kinds of MCMC samplers involved, including three kinds of scalar samplers and two kinds of multivariate (block) samplers (Roberts & Sahu, 1997; Sargent, Hodges, & Carlin, 2000). Here, we use “parameter” to mean any estimated quantity, random effect, latent state, or posterior dimension being sampled.

Adaptive random‐walk Metropolis–Hastings (ARWMH) samplers propose a new value for a parameter from a normal distribution centered on the current value, followed by accepting or rejecting that value according to the Metropolis–Hastings acceptance probability (Hastings, 1970; Metropolis, Rosenbluth, Rosenbluth, Teller, & Teller, 1953). The “adaptive” aspect updates the standard deviation of the proposal distribution to achieve an acceptance rate with good mixing (Haario, Saksman, & Tamminen, 1999; Roberts & Rosenthal, 2001). While simple and sometimes slow mixing per iteration, ARWMH is computationally fast, allowing it to run many iterations. Slice samplers (Neal, 2003) explore a range of new values for a parameter based on the current value. They almost always result in a new value. They may mix better than ARWMH, but they have higher computational cost due to exploring potentially many values, each requiring associated model calculations. In practical implementations, slice samplers should only be used when the conditional distribution of the parameter being sampled is unimodal, which will commonly be the case. For discrete‐valued parameters, one may achieve conjugate (Gibbs) sampling by trying every possible discrete value to determine the full conditional distribution by computation, which we call “computational Gibbs.” This also incurs model computations for each candidate value, a reason that sampling categorical variables can be slow. Discrete unimodal parameters can also be sampled with slice samplers. We also mention regular Gibbs (or “conjugate”) samplers, which draw a new value for a parameter from its conditional distribution when that distribution can be written analytically. That is only the case for certain fortunate combinations of prior and likelihood, which do not occur in the examples below.

Even generally efficient univariate samplers will mix slowly when the posterior has strong correlations among two or more parameters. The two kinds of block samplers used here are multivariate adaptive random‐walk Metropolis–Hastings samplers (“block_RW”) and automated‐factor slice samplers (“block_AFSS”, Tibbits, Groendyke, Haran, & Liechty, 2014). The block_RW sampler is like the ARWMH sampler above but draws its proposal from a multivariate normal distribution. The adaptation of this sampler attempts to find a proposal covariance that yields good mixing. The block_AFSS sampler uses univariate slice samplers in a set of orthogonal rotated coordinates, determined by adaptation as the MCMC gains information on the posterior. Like their univariate counterparts, the block_RW may mix more slowly per iteration but compute more quickly (allowing more iterations) than the block_AFSS.

NIMBLE and JAGS make different default sampler choices. Both assign a Gibbs sampler where possible, but the examples here do not have conjugate relationships suitable for Gibbs sampling. For nonconjugate continuous‐valued parameters, NIMBLE's default sampler assignment is an adaptive random‐walk Metropolis–Hastings sampler. In contrast, JAGS assigns a slice sampler for continuous‐valued parameters when possible. For the discrete‐valued parameters, NIMBLE assigns a computational Gibbs sampler for binary‐valued or categorical parameters and a slice sampler for parameters with more than two possible states and unimodal posterior. In contrast, JAGS assigns computational Gibbs samplers for discrete‐valued parameters with finite support (e.g., binomial distributions) and slice samplers for discrete‐valued parameter with infinite support (e.g., Poisson distributions). With JAGS, there is little user control over samplers, while NIMBLE views its defaults as just the first choices that a user can and regularly should easily modify.

Using these samplers, we chose a set of sampling strategies for comparisons for each model. These included the default samplers for NIMBLE (“nimble”) and JAGS (“jags”), the default JAGS strategy run in NIMBLE (“jags_like_nimble”), and blocking selected parameters using block_RW or block_AFSS while sampling remaining parameters using NIMBLE's default samplers.

### 2.6 Block sampling in MCMC

To block parameters, we examined each model and formulate strategies based on possible correlations between the parameters (Table 1). There are many ways one might consider blocking parameters. We limited ourselves to one set of blocking choices for each model, based on preliminary explorations. Our goal was not to determine the absolute best blocking strategy but rather to use a reasonable strategy for each model. These serve to illustrate how blocking can compare to other methods. For the single‐season multispecies occupancy model, we blocked persistence and colonization parameters for each year, yielding multiple two‐dimensional parameter blocks. For the two multispecies occupancy models, we blocked species‐specific slopes and intercepts, yielding as many parameter blocks as there are species. For the N‐mixture model, we blocked the intercept and slopes of covariates, yielding a single block.

### 2.7 Prior distributions

For most parameters, we used uninformative priors of nearly flat normal distributions for the means of the distributions of the top‐level parameters, and uniform priors over the interval [0,100] for standard deviations (see Appendix 1 for specific priors for each model). For the N‐mixture model, we followed the example of Kéry and Royle (2016) and used narrower prior distributions (Appendix 1.4).

### 2.8 Comparing MCMC efficiency

To compare performance, we look at MCMC efficiency, which we define for each parameter as the effective sample size (ESS) divided by computation time (number of effectively independent samples per second). The effective sample size gives the equivalent number of independent samples that would contain the same statistical information as the actual nonindependent samples. For a single metric of MCMC performance, we use the minimum MCMC efficiency across all the parameters because the slowest mixing parameter limits the validity of results. Computation time is measured for the actual MCMC runs, not the steps to prepare for a run, because the latter has more to do with rote software engineering than with the algorithms of interest. To translate MCMC efficiency into practical terms, we convert MCMC efficiency to the time required for sampling strategies to generate 1,000 effectively independent samples for the slowest mixing parameter (1,000/efficiency is the time in seconds to generate 1,000 effectively independent samples). For example, to generate 1,000 effectively independent samples, efficiencies of 0.01, 1, 100, and 1,000, require waiting 1.2 days, 16.7 min, 10 s, and 1 s, respectively.

All methods were run for 300,000 posterior samples. In some cases, a much smaller sample would be adequate for analysis, but larger samples support more accurate estimation of ESS for the comparisons here. We used a combination of Geweke statistics (Geweke, 1992) and visual examination of the chains to determine convergence. For comparison purposes, we did not thin samples. Although thinning can be an important practical step, it clouds comparison of MCMC performance because it always entails a loss of information (MacEachern & Berliner, 1994). Thus, to simplify comparisons, we always compare unthinned samples. R code to run all of the models and MCMC algorithms are available at https://github.com/lponisio/hierarchical, https://doi.org/10.5281/zenodo.3583426. Analyses were conducted in R 3.6.1 (R Core Team, 2017) and NIMBLE *v*0.71.

## 3 RESULTS

**Figure A1:**
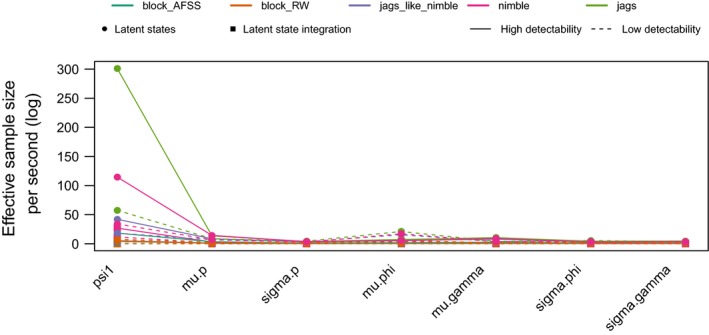
The parameter efficiency of the more hierarchical single‐species, multiseason occupancy model (Eq. A1). Colors denote the different sampling strategies. In the models, latent states were either sampled (circled) or integrated out (squares). Data were either generated with high detectability (solid line) or low detectability (dashed line)

**Figure A2:**
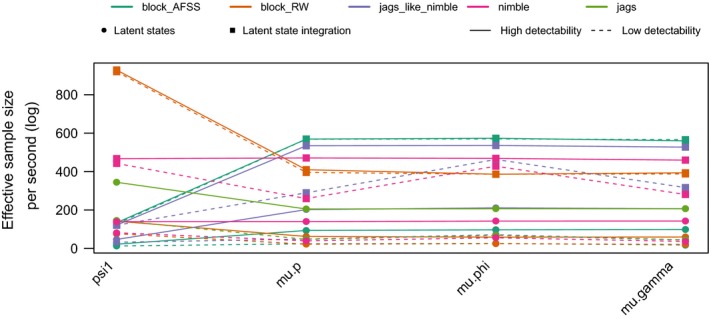
The parameter efficiency of the less hierarchical single‐species, multiseason occupancy model (Eq. A2). Colors denote the different sampling strategies. In the models, latent states were either sampled (circled) or integrated out (squares). Data were either generated with high detectability (solid line) or low detectability (dashed line)

**Figure A3:**
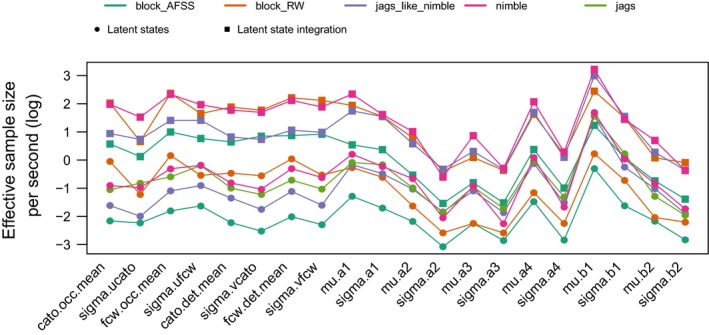
The parameter efficiency of the more hierarchical multispecies, single‐season occupancy model (Eqs. (A3), (A4). Colors denote the different sampling strategies. In the models, latent states were either sampled (circled) or integrated out (squares)

**Figure A4:**
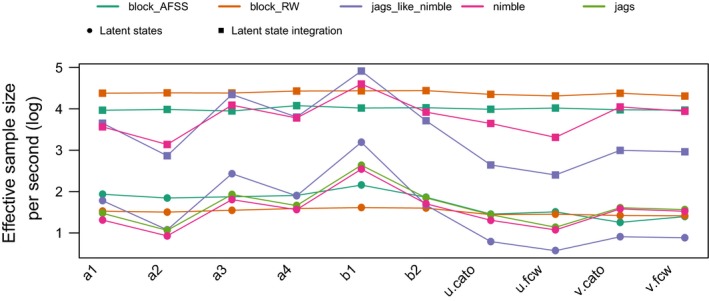
The parameter efficiency of the less hierarchical multispecies, single‐season occupancy model (Eq. A5). Colors denote the different sampling strategies. In the models, latent states were either sampled (circled) or integrated out (squares)

**Figure A5:**
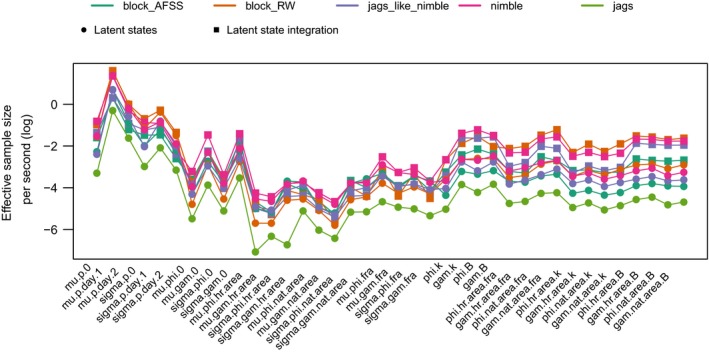
The parameter efficiency of the more hierarchical multispecies, multiseason occupancy model (Eqs. (A6), (A7), (A8), (A9), (A10). Colors denote the different sampling strategies. In the models, latent states were either sampled (circled) or integrated out (squares)

**Figure A6:**
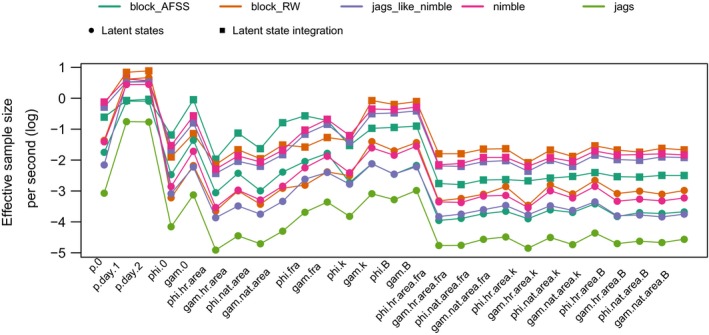
The parameter efficiency of the less hierarchical multispecies, multiseason occupancy model (Eq. A11). Colors denote the different sampling strategies. In the models, latent states were either sampled (circled) or integrated out (squares)

All chains converged sufficiently, and all posteriors from different methods for the same model scenario were in agreement (Appendix 2: Table A1, A2, A3, A4, A5, A6, A7, A8). Interestingly, the slowest mixing parameters were generally consistent across MCMC strategies (Appendix 3: Figures A1, A2, A3, A4, A5, A6), suggesting different strategies did not have strong effects on the relative sampling efficiency of specific parameters. Across all occupancy and N‐mixture models, efficiency was always much higher in the models without additional hierarchy (species‐, year‐, site‐, survey‐specific parameters). As expected, latent state integration and MCMC samplers did not have consistent effects on efficiency across models.

### 3.1 Occupancy: Single‐species, multiseason model

**Figure 1:**
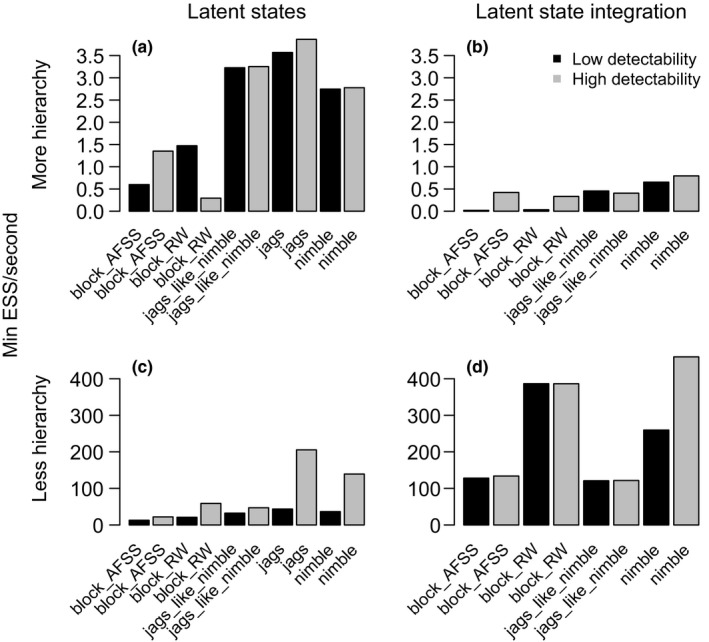
Results for the single‐species, multiseason occupancy model, showing minimum efficiency for each MCMC sampling strategy. Efficiency is defined as the effective sample size (ESS) per second. Higher efficiency is better. The model with more hierarchy includes year‐specific parameters drawn from a common distribution for year‐specific persistence and colonization probabilities. To integrate out the latent states, we use a hidden Markov model probability summation across the discrete latent state across all times for a given site

For the single‐species, multiseason example, there were interactions between the model hierarchical structure, integrating over latent states, and sampling strategy (Figure 1). With more hierarchical structure, integrating over latent states decreased efficiency compared to sampling latent states (1 min in comparison with 5.5 min to generate 1,000 effectively independent samples using default NIMBLE, Figure 1a,b). In contrast, with a less hierarchical model, integrating over latent states improved efficiency, though all sampling strategies were very efficient (only a few seconds to generate 1,000 independent samples regardless of the MCMC approach, Figure 1c,d). When latent states were sampled, JAGS, JAGS‐like NIMBLE, and default NIMBLE performed similarly in the more hierarchical model (Figure 1a,b), but JAGS had the highest efficiency in the less hierarchical model (Figure 1c).

The low detectability generally decreased sampling efficiency (Figure 1), though in some cases it did not affect efficiency (Figure 1a,b for NIMBLE and JAGS). In this simple model, sampling additional latent states may not decrease efficiency detectably. In addition, a lower detection probability may have increased posterior correlations that would decrease efficiency in some but not all MCMC strategies.

### 3.2 Occupancy: Multispecies, single‐season model

**Figure 2:**
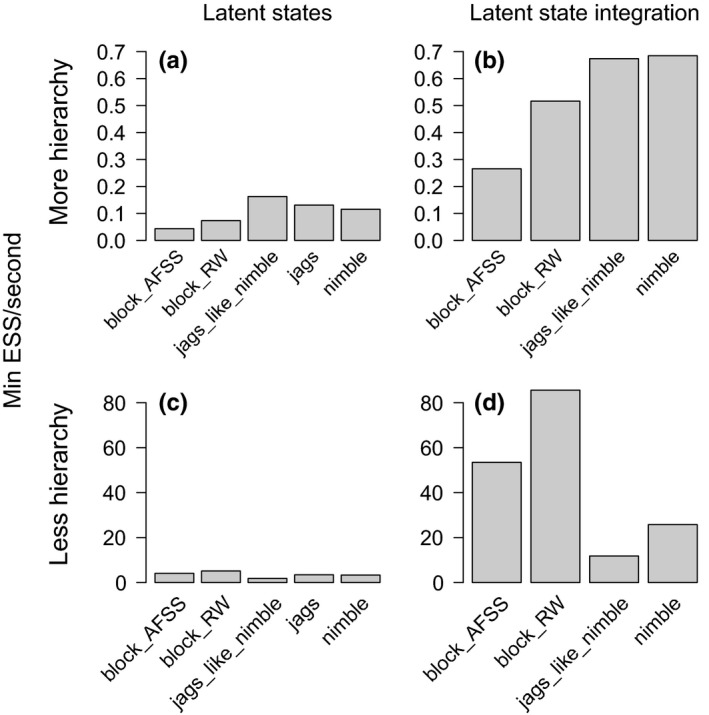
Results for the multispecies, single‐season occupancy model of bird communities, showing minimum efficiency for each MCMC sampling strategy. In the more hierarchical model, we include species‐specific coefficients and their hyperparameters for the effect of habitat characteristics on occupancy and detection. To integrate out latent states, we sum over the possible occupancy states for each species at each site

Model structure and size interacted with the sampling strategies to determine efficiency in the multispecies, single‐season example (Figure 2), but in ways that differed from the single‐species, multiseason occupancy models. In this case, integrating over latent states improved efficiency regardless of model hierarchy (the difference between 2.4 hr and 24 min to generate 1,000 independent samples using default NIMBLE in the more hierarchical model, and 3.2 min and 11 s in the less hierarchical model with adaptive random‐walk Metropolis–Hastings block sampling of some parameters; Figure 2, compare a & b and c & d).

When latent states are sampled, block sampling decreased efficiency in the more hierarchical model (Figure 2a) but increased efficiency in the less hierarchical model (Figure 2c). Moreover, block sampling also increased efficiency when latent states were integrated (Figure 2b, d), especially in the less hierarchical model (Figure 2d). In addition, for the less hierarchical model, block sampling yielded much less variation across parameters in ESS, whereas other methods yielded large differences between fast‐mixing and some slow‐mixing parameters (Appendix 3: Figure A4). When latent states are sampled, JAGS and JAGS‐like NIMBLE again perform similarly, a little better than NIMBLE's default samplers (Figure 2a, c), but only NIMBLE supports the efficiency of integrating over latent states in this example.

### 3.3 Occupancy: Multispecies, multiseason model

**Figure 3:**
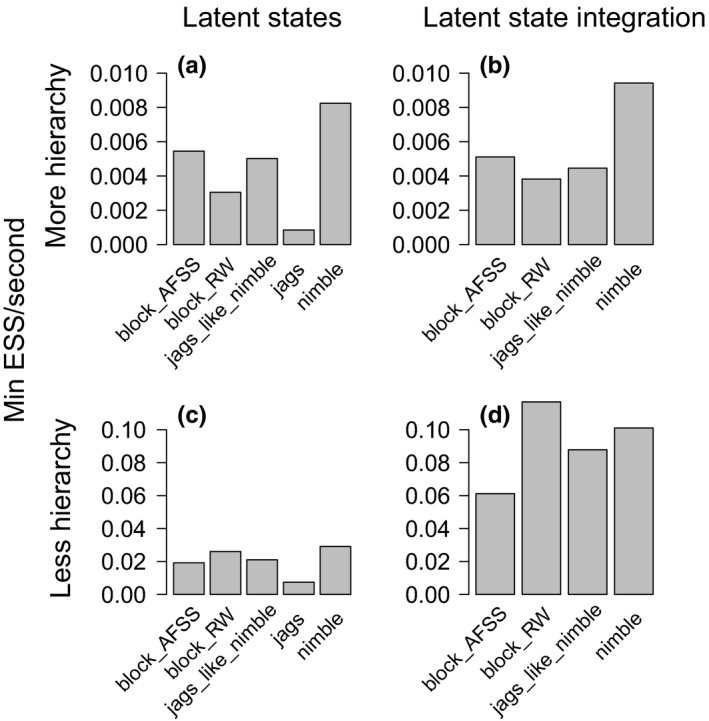
Results for the multispecies, multiseason occupancy model of bee communities, showing minimum efficiency for each MCMC sampling strategy. The more hierarchical model includes species‐specific coefficients drawn from common distributions for habitat effects on persistence and colonization. To integrate out the latent states, we sum the latent states through time for a species at a site

For the multispecies, multiseason example, integrating over latent states in the more hierarchical model yielded only minor efficiency changes compared to sampling latent states (Figure 3a, b). In contrast, integrating over latent states in the less hierarchical model was more efficient than sampling latent states (the difference between 9.5 and just under 3 hr to generate 1,000 independent samples in default NIMBLE, Figure 3c,d). When sampling latent states, JAGS performed much worse than any of the NIMBLE configurations (the difference between 14.5 d and 1.2 d to generate 1,000 independent samples in the more hierarchical model, Figure 3a,c). The default NIMBLE samplers tended to have the highest efficiency across model structures and sizes (Figure 3). One exception is that in the model where latent states are integrated with the less hierarchical model, the random‐walk block has slightly higher efficiency (Figure 3d).

### 3.4 N‐mixture model: Zero‐inflated Poisson

**Figure 4:**
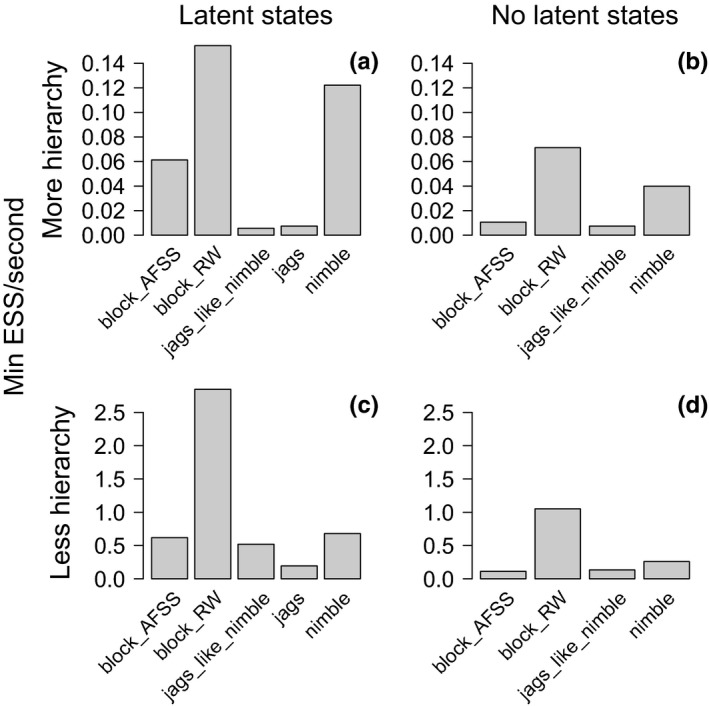
Results for the N‐mixture model of Swiss great tits, showing minimum efficiency for each MCMC sampling strategy. In the more hierarchical model, we included site‐ and survey‐specific parameters and their hyperparameter distributions on both abundance and detection. To integrate out latent states, we sum over the range of values of N, the true local abundance at each site

**Figure A7:**
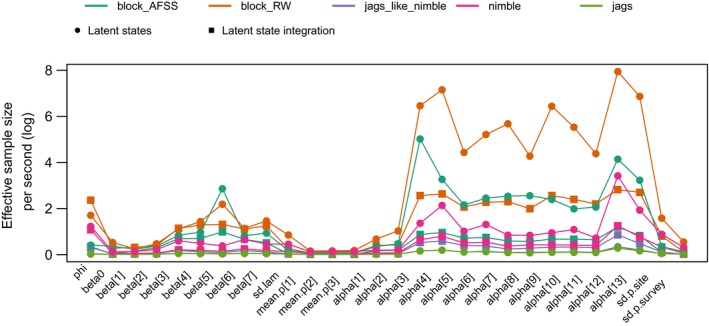
The parameter efficiency of the more hierarchical zero‐inflated N‐mixture model (Eqs. (A12), (A13), (A14). Colors denote the different sampling strategies. In the models, latent states were either sampled (circled) or integrated out (squares)

**Figure A8:**
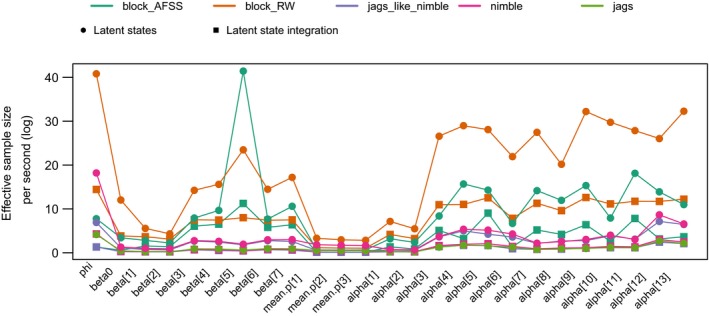
The parameter efficiency of the more hierarchical zero‐inflated N‐mixture model. Colors denote the different sampling strategies. In the models, latent states were either sampled (circled) or integrated out (squares)

In the N‐mixture example, integrating over latent states is generally less efficient than sampling latent states, regardless of more vs. less hierarchy (the difference between just under an hour and over four hours to generate 1,000 independent samples using default NIMBLE in the more hierarchical model, Figure 4). This is likely because the summation over a large range of possible N values is computationally costly. We, therefore, focus on comparing MCMC strategies where latent states are sampled. Random‐walk block sampling had the highest efficiency across all model sizes and structures. JAGS and JAGS‐like NIMBLE had the lowest efficiencies across all models (the difference between 16 hr in JAGS and 30 min using NIMBLE and the random‐walk block sampler, Figure 4). The slowest mixing parameters were generally consistent across MCMC strategies (Appendix 3: Figures A7, A8).

## 4 DISCUSSION

Our results highlight that the best strategy for MCMC depends on the model. Because of interactions between the handling of latent states (direct integration vs. sampling), model structure, model type (single‐ vs. multiseason and single‐ vs. multispecies cases of occupancy models; and N‐mixture models), and sampling strategy, there are no one‐size‐fits‐all best strategies for MCMC. These results accord with typical results from the statistical literature that different strategies work well for different models (e.g., Browne et al., 2009; Solonen et al., 2012). efficient. Furthermore, efficiency varies over many orders of magnitude for different cases.

An example of the context dependence of MCMC strategies is that latent state integration improved efficiency drastically in some models (both multispecies occupancy models), while it reduced efficiency just as strongly in others (the more hierarchical single‐species, multiseason occupancy model and the N‐mixture model). This difference in results likely arises because the computational cost of latent state integration is different for each model. For the single‐season occupancy model, marginalization is a quick summation over the two possibilities of true occupancy or not for a given site. Hence, it is computationally efficient and removes the need to sample those latent states, yielding a net benefit. For the multiseason occupancy models, marginalization is a hidden Markov model filtering calculation that sums over the two possibilities states sequentially through time for a given site, which is much more costly. This may explain the decrease in MCMC efficiency when integrating over latent states in this model. For the multiseason, multispecies model, NIMBLE's efficiency (with default samplers) was similar when integrating vs. sampling latent states. This may reflect a balance between the benefits and costs revealed by the two simpler cases. However, additional factors such as the overall complexity of the larger model may also play a role. For the N‐mixture model, it seems clear that the cost is much higher than any benefit of integrating over latent states. Our model efficiency comparisons for occupancy and N‐mixture models suggest that integrating over latent states seems to be beneficial primarily when such an integration is simple and efficient.

The costs and benefits of block sampling will also be different for different model structures. In our examples, the best gain from block sampling occurred for the more hierarchical N‐mixture model when sampling latent states. When there are strong correlations in some dimensions of the posterior, while others are held fixed, then block sampling has the potential to improve mixing. However, in random‐walk block sampling, many proposals are rejected, and adaptation to an efficient proposal covariance can be slow, with both issues being more severe when more dimensions are jointly sampled. In the automated‐factor slice sampler, a slice sampler is applied in each of a set of orthogonal coordinates that combine multiple parameters. While this can yield good mixing, its poor performance can be attributed to its high computational cost; slice sampling involves many model likelihood calculations. Another important trade‐off in block sampling is whether the parameters being jointly sampled require the same components of the model to be calculated for the MCMC update. If the parameters being jointly sampled do not share the same model components, the MCMC update will have a higher computational cost. There are many approaches to block sampling, and, more generally, sampling correlated dimensions of the posterior. The results here include basic ideas and establish that the success of block sampling, like other aspects of MCMC, depends on the model.

An important caveat with our analysis is that the efficiency of MCMC depends on which parameterization is used and which results are of interest. We have emphasized the MCMC efficiency of the worst‐mixing parameter because one should be cautious about any output unless all parameters are well mixed. This means that the quality of results is limited by the worst‐mixing parameter. However, if one decides that, say, log of a standard deviation instead of standard deviation is the parameter of interest, one may obtain different results. This is life in a Bayesian framework. Bayesian results are not invariant to parameterization in general, and, specifically, the effective sample size of a nonlinear function of a posterior sample will not match that of the sample itself. Such issues are not likely to completely flip or even qualitatively change the outcome of comparisons such as ours, but they would quantitatively change them to some degree.

Though MCMC efficiency was context‐dependent, some general patterns emerged. First, it is almost always possible to obtain large boosts in MCMC efficiency from some customization of the model and sampling strategy compared to simple default approaches. The largest boosts would vastly change a user's model‐fitting experience—for example, the difference between 2 weeks in JAGS and just over a day in NIMBLE to generate 1,000 independent samples for the multispecies, multiseason model. The only case where one of the default strategies was best was the more hierarchical single‐species, multiseason occupancy model where latent states were sampled. Our results are consistent with, but less extreme than, those of Turek et al. (2016), who investigated the use of NIMBLE for capture–recapture models. Their most extreme efficiency gain was almost an 1,000‐fold improvement for a large multistate capture model. Compared to results here, their larger improvement may be attributable to higher model complexity, use of the automated blocking algorithm of Turek et al. (2017), and more detailed customization efforts.

Second, the most meaningful improvements were possible with the most complex (slowest mixing) examples, where the type of gains possible could mean the difference between a week and a day, or a day and an hour, of computation. Third, including more hierarchical structure always slows mixing, a fact well known to practitioners. Fourth, simpler sampling strategies sometimes outperform more advanced strategies if the former are computationally cheaper than the latter and so can iterate more quickly. Finally, customization of the model and sampling strategy with NIMBLE yielded substantial performance improvements over JAGS in all but one case (Figure 1a), where they were essentially tied. In the two more complicated models (multispecies, multiseason occupancy, and N‐mixture), the default performance of NIMBLE was 2.5×–10× more efficient than JAGS, and the best performance was 5×–12× more efficient. Given that JAGS [and other software in the BUGS language, Surhone et al., 2010; Lunn, Thomas, Best, & Spiegelhalter, 2000) is widely used and has been transformative in the practice of Bayesian hierarchical modeling in ecology, our results suggest that NIMBLE can be much more efficient than JAGS in the cases where it matters most, when overall efficiency is quite low.

## CONFLICT OF INTEREST

None Declared.

## AUTHOR CONTRIBUTIONS

LCP and PD designed the study. PD designed the blocking strategies and functions for latent state integration. LCP implemented and ran the models and MCMC customizations. LCP, PD, DT, and NM contributed to coding and debugging. LCP made the tables and figures. LCP and PD interpreted the results with input from DT. LCP and PD wrote the first draft of the manuscript, and all other authors contributed to revisions. Publication made possible in part by support from the Berkeley Research Impact Initiative (BRII) sponsored by the UC Berkeley Library.

### Open Research Badges

This article has been awarded Open Materials, Open Data Badges. All materials and data are publicly accessible via the Open Science Framework at https://github.com/lponisio/hierarchical; https://doi.org/10.5281/zenodo.3583426.

## Data Availability

Data are deposited in GitHub with the analysis code at https://github.com/lponisio/hierarchical or https://doi.org/10.5281/zenodo.3583426

## APPENDIX 1

### MODEL DETAILS 1

Notation varies across examples. Parameter labels used in tables and figures correspond to variable names in the code, which is generally related to but not the same as the mathematical notation of each model's description.

#### Occupancy: Single‐species, multiseason model

The single‐species multiseason occupancy model (i.e., dynamic occupancy model, Royle & Kéry, 2007) uses simulations modified from Kery and Schaub (2012). We simulated 100 sites sampled 5 times per year for 15 years. Let *z_i,j_* denote the true occupancy of a species at the *i* ^th^ site in the *j* ^th^ year. *z_i,j_* is a Bernoulli random variable, *z_i,j_*~ Bern(*ψ_i,j_*). In the first year, *z_i,_*_1_ ~ Bern(*ψ_i_*_,1_). *ψ_i_*_,1_ is the occupancy probability at the *i* ^th^ site in the *j* ^th^ year.

The model with more hierarchical structure includes year‐specific persistence and colonization probabilities, each from a prior distribution. Let *ϕ_j_* denote the logit probability the species persists at a site from years *j* to *j* + 1 (given that *z_i,j_ = *1) and *γ_j_* denote the logit probability that site *i* is colonized in year *j* + 1 (given that *z_i,j_ = *0). The priors for *γ_j_* and *ϕ_j_*, priors for their hyperparameters, and the calculation of *ψ_i,j_* are given by:

(A1) $$\begin{array}{cc} \mu_{\gamma } & ∼N\left(0,10^{6}\right)\\ \mu_{\phi } & ∼N\left(0,10^{6}\right)\\ \sigma_{\gamma } & ∼\text{uniform}\;\left(0,100\right)\\ \sigma_{\phi } & ∼\text{uniform}\;\left(0,100\right)\\ \gamma_{j} & ∼N\left(\mu_{\gamma },\sigma_{\gamma }^{2}\right)\\ \phi_{j} & ∼N\left(\mu_{\phi },\sigma_{\phi }^{2}\right)\\ \psi_{i,1} & ∼\text{uniform}\;\left(0,1\right)\\ \psi_{i,j+1} & =\;\text{expit}\;\left(\phi_{j}\right)*z_{i,j}+\;\text{expit}\;\left(\gamma_{j}\right)*\left(1-z_{i,j}\right) \end{array}$$

Here, expit is the inverse of the logit function: expit (z) = 1/(1 + *e* ^−^ *^z^*). Note that, since *z_i,j_* is either 0 or 1, it is equivalent to write $\text{logit}\;\left(\psi_{i,j+1}\right)=\phi_{j}\times z_{i,j}+\gamma_{j}\;\times \;\left(1-z_{i,j}\right)$. Our notation for normal distributions uses mean and variance, that is, “*N* (mean, variance)”.

We then let *y_i,j,k_* indicate whether the species was (*y_i,j,k_* = 1) or was not (*y_i,j,k_* = 0) detected in the *k* ^th^ visit to site *i* in year *j*. The logit probability of detection when a site is occupied in year *j* is *p_j_*. Similar to *γ_j_* and *ϕ_j_*, *p_j_* is year‐specific and follows a normal distribution. These relationships and relevant priors are given as follows:

(A2) $$\begin{array}{cc} \mu_{p} & ∼N\left(0,10^{6}\right)\\ \sigma_{p} & ∼\text{uniform}\;\left(0,100\right)\\ p_{j} & ∼N\left(\mu_{p},\sigma_{p}^{2}\right)\\ y_{i,j,k} & ∼\text{Bern}\left(\;\text{expit}\;\left(p_{j}\right)*z_{i,j}\right) \end{array}$$

The model with less hierarchical structure lacks the year‐specific parameters; instead, there are single, time‐independent *γ*, *ϕ*, and *p* parameters, so their subscripts *j* can be removed in the above equations. Each follows a normal prior with mean 0 and variance 10^6^. In this case, there is no need for *µ_γ_*, *σ_γ_*, *µ_ϕ_*, *σ_ϕ_*, *µ_p_*, or *σ_p_*.

#### Occupancy: Multispecies, single‐season model

The multispecies, single‐season occupancy example models bird community data in relation to variables about wildlife management and habitat characteristics (Zipkin et al., 2010). There are 70 sites, each sampled 3–4 times for detection/nondetection of 58 species. For species i and site j, the probability of occupancy is $\psi_{i,j}$ and the true occupancy status is $z_{i,j}$, that is, $z_{i,j}∼\text{Bern}\left(\psi_{i,j}\right)$. Detection probability for the $k^{\text{th}}$ visit to site *j* for species *i* is $p_{i,j,k}$. The corresponding detection/nondetection datum is $y_{i,j,k}∼\text{Bern}\left(p_{i}*z_{i,j}\right)$.

Both site occupancy and detection were allowed to depend on habitat and study area (Zipkin et al., 2010). The two study areas were labeled CATO (Catoctin Mountain Park) and FCW (Frederick City Watershed Cooperative Wildlife Management Area), with indicator variable ${\;\text{Ind}\;}_{j}$ set to 0 or 1 if site *j* was in the CATO or FCW study area, respectively. The two study areas corresponded to different deer management strategies.

Occupancy probability was allowed to depend on study area, tree basal area (BA), and understory foliage cover (UFC). Detection probability was allowed to depend on study area and date. Relationships with BA, UFC, and date included both linear and quadratic terms.

In the model with more hierarchical structure, the coefficients of BA, UFC, and study area are species‐specific and drawn from common distributions with similar priors as above:

(A3) $$\begin{array}{cc} \text{expit}\;\left(\mu_{\;\text{uCATO}\;}\right) & ∼\text{uniform}\;\left(0,1\right)\\ \text{expit}\;\left(\mu_{\;\text{uFCW}\;}\right) & ∼\text{uniform}\;\left(0,1\right)\\ \sigma_{\;\text{uCATO}\;} & ∼\text{uniform}\;\left(0,100\right)\\ \sigma_{\;\text{uFCW}\;} & ∼\text{uniform}\;\left(0,100\right)\\ \mu_{a1} & ∼N\left(0,10^{6}\right)\\ \mu_{a2} & ∼N\left(0,10^{6}\right)\\ \mu_{a3} & ∼N\left(0,10^{6}\right)\\ \mu_{a4} & ∼N\left(0,10^{6}\right)\\ \sigma_{a1} & ∼\text{uniform}\;\left(0,100\right)\\ \sigma_{a2} & ∼\text{uniform}\;\left(0,100\right)\\ \sigma_{a3} & ∼\text{uniform}\;\left(0,100\right)\\ \sigma_{a4} & ∼\text{uniform}\;\left(0,100\right)\\ {\text{uCATO}\;}_{i} & ∼N\left(\mu_{\;\text{uCATO}\;},\sigma_{\;\text{uCATO}\;}^{2}\right)\\ {\text{uFCW}\;}_{i} & ∼N\left(\mu_{\;\text{uFCW}\;},\sigma_{\;\text{uFCW}\;}^{2}\right)\\ a1{}_{i} & ∼N\left(\mu_{a1},\sigma_{a1}^{2}\right)\\ a2{}_{i} & ∼N\left(\mu_{a2},\sigma_{a2}^{2}\right)\\ a3{}_{i} & ∼N\left(\mu_{a3},\sigma_{a3}^{2}\right)\\ a4{}_{i} & ∼N\left(\mu_{a4},\sigma_{a4}^{2}\right)\\ \text{logit}\left(\psi_{i,j}\right) & ={\text{uCATO}\;}_{i}*\left(1-{\;\text{Ind}\;}_{j}\right)+{\;\text{uFCW}\;}_{i}{\text{* Ind}\;}_{j}\\ & \;+a1{}_{i}{\text{* UFC}\;}_{j}+a2{}_{i}{\text{* UFC}\;}_{j}^{2}+a3{}_{i}{\text{* BA}\;}_{j}+a4{}_{i}{\text{* BA}\;}_{j}^{2} \end{array}$$

Similarly, the species‐specific detection probabilities are modeled with species‐specific coefficients from distributions with priors, all as follows:

(A4) $$\begin{array}{cc} \text{expit}\left(\mu_{\text{vCATO}}\right) & ∼\text{uniform}\left(0,1\right)\\ \text{expit}\left(\mu_{\text{vFCW}}\right) & ∼\text{uniform}\;\left(0,1\right)\\ \sigma_{\text{vCATO}} & ∼\text{uniform}\left(0,100\right)\\ \sigma_{\text{vFCW}} & ∼\text{uniform}\left(0,100\right)\\ \mu_{b1} & ∼N\left(0,10^{6}\right)\\ \mu_{b2} & ∼N\left(0,10^{6}\right)\\ \sigma_{b1} & ∼\text{uniform}\left(0,100\right)\\ \sigma_{b2} & ∼\text{uniform}\left(0,100\right)\\ {\text{vCATO}}_{i} & ∼N\left(\mu_{\text{vCATO}},\sigma_{\text{vCATO}}^{2}\right)\\ {\text{vFCW}}_{i} & ∼N\left(\mu_{\;\text{vFCW}},\sigma_{\text{vFCW}\;}^{2}\right)\\ b1_{i} & ∼N\left(\mu_{b1},\sigma_{b1}^{2}\right)\\ \text{logit}\left(p_{i,j,k}\right) & ={\text{vCATO}}_{i}*\left(1-{\text{Ind}}_{j}\right)+{\text{vFCW}\;}_{i}*{\text{Ind}}_{j}\\ & \;+b1_{i}*{\text{date}}_{j}+b2_{i}*{\text{date}}_{j}^{2} \end{array}$$

The case with less hierarchical structure has no species‐specific parameters, so all of the species share the same coefficients for the effect of habitat and management. This gives:

(A5) $$\begin{array}{cc} \text{logit}\left(\psi_{i,j}\right) & =\text{uCATO *}\left(1-{\;\text{Ind}\;}_{j}\right)+{\;\text{uFCW * Ind}\;}_{j}\\ & \;+a1{\text{* UFC}\;}_{j}+a2{\text{* UFC}\;}_{j}^{2}+a3{\text{* BA}\;}_{j}+a4{\text{* BA}\;}_{j}^{2}\\ \text{logit}\left(p_{i,j,k}\right) & =\text{vCATO *}\left(1-{\;\text{Ind}\;}_{j}\right)+{\;\text{vFCW * Ind}\;}_{j}\\ & \;+b1{\text{* date}\;}_{j}+b2{\text{* date}\;}_{j}^{2} \end{array}$$

As for the first example, the priors for the coefficients in this model are normal with mean 0 and standard deviation 1,000. The purpose of this model is not to be a scientific alternative to the more hierarchical model but rather to provide a useful case for comparison of MCMC methods.

#### Occupancy: Multispecies, multiseason model

The multispecies, multiseason occupancy example models data on wild bees in on‐farm habitat restoration patches (Ponisio et al., 2019). There are 31 sites, each sampled 2–7 times per year for 10 years for detection/nondetection of 49 bee species. For species *i*, site *j*, and year *t*, the latent occupancy state is *z_i,j,t_*. For the same indices and visit *r*, the detection probability is *p_i,j,k,r_*, and the detection/nondetection datum is $y_{i,j,t,r}$. Thus, $y_{i,j,t,r}∼\text{Bern}\left(p_{i,j,t,r}*z_{i,j,t}\right)$.

Occupancy probability is denoted $\psi_{i,j,t}$ and defined by persistence and colonization probabilities. If a site is occupied in year *t*, the logit probability that it persists (continues to be occupied) in year *t* + 1 is $\phi_{i,j,t}$. If a site is unoccupied in year *t*, the logit probability that it is colonized (becomes occupied) in year *t* + 1 is $\gamma_{i,j,t}$. The definition of $\psi_{i,j,t}$ is then given by:

(A6) $$\text{logit}\;\left(\psi_{i,j,t+1}\right)=\phi_{i,j,t}*z_{i,j,t}+\gamma_{i,j,t}*\left(1-z_{i,j,t}\right)$$

Note that only one term of the right‐hand side will be nonzero.

Probability of occupancy in year 1 is denoted $\psi_{i,j,1}$ and defined as the equilibrium occupancy calculated from the mean logit persistence and colonization probabilities. Specifically,

(A7) $$\begin{array}{cc} \text{logit}\;\left({\bar{\phi }}_{i,j,\cdot }\right) & =\frac{1}{9}\sum_{t=1}^{9}\phi_{i,j,t}\\ \text{logit}\;\left({\bar{\gamma }}_{i,j,\cdot }\right) & =\frac{1}{9}\sum_{t=1}^{9}\gamma_{i,j,t}\\ \psi_{i,j,1} & =\frac{{\bar{\gamma }}_{i,j,\cdot }}{1-{\bar{\phi }}_{i,j,\cdot }-{\bar{\gamma }}_{i,j,\cdot }} \end{array}$$

Site‐level intercept and slope parameters for the effects of habitat quality (floral resource diversity, ${\;\text{frd}\;}_{j,t}$) and the weighted proximity of other habitat patches (hedgerows and remnant natural habitat, ${\;\text{HRwtprox}\;}_{j}$ and ${\;\text{RemnantWtProx}\;}_{j}$, respectively) were modeled as independent species‐level random effects. Species‐level covariates included species' body size (${\;\text{BodySize}\;}_{i}$) and diet breadth ($k_{i}$). Interactions between site‐level and species‐level covariates were also included. The persistence components of the model including priors are as follows:

(A8) $$\begin{array}{cc} \mu_{\alpha } & ∼N\left(0,10^{6}\right)\\ \mu_{\beta \left[s\right]} & ∼N\left(0,10^{6}\right),s=1\ldots 3\\ \sigma_{\alpha } & ∼\text{uniform}\;\left(0,100\right)\\ \sigma_{\beta \left[s\right]} & ∼\text{uniform}\;\left(0,100\right),s=1\ldots 3\\ \alpha_{i} & ∼N\left(\mu_{\alpha },\sigma_{\alpha }^{2}\right)\\ \beta {\left[s\right]}_{i} & ∼N\left(\mu_{\beta \left[s\right]},\sigma_{\beta \left[s\right]}^{2}\right),s=1\ldots 3\\ \beta \left[s\right] & ∼N\left(0,10^{6}\right),s=4\ldots 10\\ \phi_{i,j,t} & =\alpha_{i}+\beta {\left[1\right]}_{i}{\text{*HRwtProx}}_{j}+\beta {\left[2\right]}_{i}{\text{*RemnantWtProx}}_{j}+\beta {\left[3\right]}_{i}{\text{*frd}}_{j,t}\\ & \;+\beta \left[4\right]{\text{*k}}_{i}+\beta \left[5\right]{\text{*BodySize}}_{i}\\ & \;+\beta \left[6\right]{\text{*frd}}_{j,t}{\text{*HRwtProx}}_{j}+\beta \left[7\right]{\text{*frd}}_{j,t}{\text{*RemnantWtProx}}_{j}\\ & \;+\beta \left[8\right]{\text{*k}}_{i}{\text{*HRwtProx}}_{j}+\beta \left[9\right]{\text{*BodySize}}_{i}{\text{*HRwtProx}}_{j}\\ & \;+\beta \left[10\right]{\text{*k}}_{i}{\text{*RemnantWtProx}}_{j}+\beta \left[11\right]{\text{*BodySize}}_{i}{\text{*RemnantWtProx}}_{j} \end{array}$$

The colonization components of the model including priors are as follows:

(A9) $$\begin{array}{cc} \mu_{A} & ∼N\left(0,10^{6}\right)\\ \mu_{B\left[s\right]} & ∼N\left(0,10^{6}\right),s=1\ldots 3\\ \sigma_{A} & ∼\text{uniform}\;\left(0,100\right)\\ \sigma_{B\left[s\right]} & ∼\text{uniform}\;\left(0,100\right),s=1\ldots 3\\ A_{i} & ∼N\left(\mu_{A},\sigma_{A}^{2}\right)\\ B{\left[s\right]}_{i} & ∼N\left(\mu_{B\left[s\right]},\sigma_{B\left[s\right]}^{2}\right),s=1\ldots 3\\ \gamma_{i,j,t} & =A_{i}+B{\left[1\right]}_{i}{\text{*HRwtProx}}_{j}+B{\left[2\right]}_{i}{\text{*RemnantWtProx}}_{j}+B{\left[3\right]}_{i}{\text{*frd}}_{j,t}\\ & \;+B\left[4\right]*k_{i}+B\left[5\right]{\text{*BodySize}}_{i}\\ & \;+B\left[6\right]{\text{*frd}}_{j,t}{\text{*HRwtProx}}_{j}+B\left[7\right]{\text{*frd}}_{j,t}{\text{*RemnantWtProx}}_{j}\\ & \;+B\left[8\right]*k_{i}{\text{*HRwtProx}}_{j}+B\left[9\right]{\text{*BodySize}}_{i}{\text{*HRwtProx}}_{j}\\ & \;+B\left[10\right]*k_{i}{\text{*RemnantWtProx}}_{j}+B\left[11\right]{\text{*BodySize}}_{i}{\text{*RemnantWtProx}}_{j} \end{array}$$

Here $\alpha_{i}$ and $A_{i}$ denote species‐specific intercepts of logit persistence and colonization probabilities, respectively. The $\beta \left[s\right]$ and *B*[*s*] parameters preceeding each of the explanatory variables represent the effect of those variables on persistence and colonization.

The detection probability of each species was allowed to vary over the season according to species‐specific phenologies defined by a quadratic function of day of year (${\;\text{date}\;}_{j,t,r}$) with species‐specific coefficients drawn from across‐species prior distributions (M'Gonigle et al., 2015). Specifically, $p_{i,j,t,r}$ was modeled as follows:

(A10) $$\begin{array}{cc} \mu_{p\left[s\right]} & ∼N\left(0,10^{6}\right),s=0\ldots 2\\ \sigma_{p\left[s\right]} & ∼\text{uniform}\left(0,100\right),s=0\ldots 2\\ p{\left[0\right]}_{i} & ∼N\left(\mu_{p\left[0\right]},\sigma_{p\left[0\right]}^{2}\right)\\ p{\left[1\right]}_{i} & ∼N\left(\mu_{p\left[1\right]},\sigma_{p\left[1\right]}^{2}\right)\\ p{\left[2\right]}_{i} & ∼N\left(\mu_{p\left[2\right]},\sigma_{p\left[2\right]}^{2}\right)\\ \text{logit}\left(p_{i,j,t,r}\right) & =p[{0]}_{i}+p[{1]}_{i}{\text{*date}}_{j,t,r}+p{\left[2\right]}_{i}{\text{*date}}_{j,t,r}^{2} \end{array}$$

where $p{\left[0\right]}_{i}$, $p{\left[1\right]}_{i}$, and $p{\left[2\right]}_{i}$ denote species‐specific intercept, linear coefficient, and quadratic coefficient, respectively, for effect of day of year on detection probability of species *i*.

The case with less hierarchical structure does not have species‐specific coefficients. Instead, all species share the same coefficients for the effect of local and landscape habitat variables. This is given by:

(A11) $$\begin{array}{cc} \phi_{i,j,k} & =\alpha +\beta \left[1\right]{\text{*HRwtProx}}_{j}+\beta \left[2\right]{\text{*RemnantWtProx}}_{j}+\beta \left[3\right]{\text{*frd}}_{j,t}\\ & \;+\beta \left[4\right]{\text{*k}}_{i}+\beta \left[5\right]{\text{*BodySize}}_{i}\\ & \;+\beta \left[6\right]{\text{*frd}}_{j,t}{\text{*HRwtProx}}_{j}+\beta \left[7\right]{\text{*frd}}_{j,t}{\text{*RemnantWtProx}}_{j}\\ & \;+\beta \left[8\right]{\text{*k}}_{i}{\text{*HRwtProx}}_{j}+\beta \left[9\right]{\text{*BodySize}}_{i}{\text{*HRwtProx}}_{j}\\ & \;+\beta \left[10\right]{\text{*k}}_{i}{\text{*RemnantWtProx}}_{j}+\beta \left[11\right]{\text{*BodySize}}_{i}{\text{*RemnantWtProx}}_{j}\\ \gamma_{i,j,t} & =A+B\left[1\right]{\text{*HRwtProx}}_{j}+B\left[2\right]{\text{*RemnantWtProx}}_{j}+B\left[3\right]{\text{*frd}}_{j,t}\\ & \;+B\left[4\right]{\text{*k}}_{i}+B\left[5\right]{\text{*BodySize}}_{i}\\ & \;+B\left[6\right]{\text{*frd}}_{j,t}{\text{*HRwtProx}}_{j}+B\left[7\right]{\text{*frd}}_{j,t}{\text{*RemnantWtProx}}_{j}\\ & \;+B\left[8\right]{\text{*k}}_{i}{\text{*HRwtProx}}_{j}+B\left[9\right]{\text{*BodySize}}_{i}{\text{*HRwtProx}}_{j}\\ & \;+B\left[10\right]{\text{*k}}_{i}{\text{*RemnantWtProx}}_{j}+B\left[11\right]{\text{*BodySize}}_{i}{\text{*RemnantWtProx}}_{j}\\ \text{logit}\left(p_{i,j,t,r}\right) & =p\left[0\right]+p\left[1\right]{\text{*date}}_{j,t,r}+p\left[2\right]*{\left({\text{date}}_{j,t,r}\right)}^{2} \end{array}$$

As for the first example, the priors for the coefficients in this model are normal with mean 0 and standard deviation 10^3^.

#### Zero‐inflated N‐mixture model

The zero‐inflated N‐mixture example models the abundance of birds (great tits) using breeding bird survey data across Switzerland (Kéry & Royle, 2016, ch. 6.11.1). There are 267 routes, each in a 1 square kilometer quadrat in a grid, each surveyed 2–3 times in one year. Kéry and Royle (2016), this example features some informative priors.

The number of birds available to be counted on each route is assumed to follow a zero‐inflated Poisson distribution. The probability of a structural zero (due to unsuitable habitat) is *ϕ*. The unobserved state $w_{i}$ is 0 if the habitat is unsuitable, 1 if it is suitable. The number of birds available to be counted at site i is $N_{i}$, which is 0 if $w_{i}=0$ and follows a Poisson with mean $\lambda_{i}$ if $w_{i}=1$. These relationships are written as follows:

(A12) $$\begin{array}{cc} w_{i} & ∼\text{Bernoulli}\left(1-\phi \right)\\ N_{i} & ∼\text{Poisson}\left(w_{i}\lambda_{i}\right) \end{array}$$

Covariates for $\lambda_{i}$ included site‐level forest cover (%, ${\;\text{forest}\;}_{i}$), elevation (m, ${\;\text{elev}\;}_{i}$), and route length (km, ${\;\text{length}\;}_{i}$). In addition, a random effect of site on $\lambda_{i}$ allowed for unexplained route‐to‐route variation. The $\lambda_{i}$ component of the model is as follows:

(A13) $$\begin{array}{cc} \beta_{0} & ∼N\left(0,10\right)\\ \beta_{s} & ∼N\left(0,1\right),s=1\ldots 7\\ \varepsilon_{\lambda ,i} & ∼N\left(0,\sigma_{\varepsilon ,\lambda }\right)\\ \sigma_{\varepsilon ,\lambda } & ∼\text{uniform}\;\left(0,2\right)\\ \log\left(\lambda_{i}\right) & =\beta_{0}+\beta_{1}{\text{* elev}\;}_{i}+\beta_{2}{\text{* elev}\;}_{i}^{2}+\beta_{3}{\text{* forest}\;}_{i}\\ & \;+\beta_{4}{\text{* forest}\;}_{i}^{2}+\beta_{5}\text{* elev * forest}\;+\beta_{6}{\text{* elev}\;}^{2}{\text{* forest}\;}^{2}+\beta_{7}{\text{* length}\;}_{i}+\varepsilon_{\lambda ,i} \end{array}$$

The observed abundance of birds on the $i^{\text{th}}$ route during the $j^{\text{th}}$ survey is then modeled, including priors, as follows:

(A14) $$\begin{array}{cc} \text{expit}\;\left(\alpha 0_{j}\right) & ∼\text{uniform}\;\left(0,1\right)\\ \alpha_{s} & ∼N\left(0,1\right),s=1\ldots 13\\ \varepsilon_{p\left(\text{site}\right),i} & ∼N\;\left(0,\sigma_{\varepsilon ,p\;\left(\text{site}\right)}^{2}\right)\\ \varepsilon_{p\left(\text{survey}\right),i,j} & ∼N\left(0,\sigma_{\varepsilon ,p\;\left(\text{survey}\right)}^{2}\right)\\ \sigma_{\varepsilon ,p\left(\text{site}\right)} & ∼\text{uniform}\;\left(0,2\right)\\ y_{i,j}|N_{i} & ∼\text{Binomial}\left(N_{i},p_{i,j}\right)\\ \text{logit}\left(p_{i,j}\right) & =\alpha 0_{j}+\alpha_{1}{\text{* elev}\;}_{i}+\alpha_{2}{\text{* elev}\;}_{i}^{2}\\ & \;+\alpha_{3}{\text{* date}\;}_{i,j}+\alpha_{4}{\text{* date}\;}_{i,j}^{2}\\ & \;+\alpha_{5}{\text{* dur}\;}_{i,j}+\alpha_{6}{\text{* dur}\;}_{i,j}^{2}\\ & \;+\alpha_{7}{\text{* elev}\;}_{i}{\text{* date}\;}_{i,j}+\alpha_{8}{\text{* elev}\;}_{i}^{2}{\text{* date}\;}_{i,j}\\ & \;+\alpha_{9}{\text{* elev}\;}_{i}{\text{* dur}\;}_{i,j}+\alpha_{10}{\text{* elev}\;}_{i}{\text{* dur}\;}_{i,j}^{2}\\ & \;+\alpha_{11}{\text{* elev}\;}_{i}^{2}{\text{* dur}\;}_{i,j}+\alpha_{12}{\text{* date}\;}_{i,j}{\text{* dur}\;}_{i,j}\\ & \;+\alpha_{13}{\text{* date}\;}_{i,j}{\text{* dur}\;}_{i,j}^{2}+\varepsilon_{p\left(\text{site}\right),i}+\varepsilon_{p\left(\text{survey}\right),i,j} \end{array}$$

where covariates for survey date (${\;\text{date}\;}_{i,j}$), duration (min, ${\;\text{dur}\;}_{i,j}$), elevation, and their interactions are included as well as random effects for site and survey.

The model with less hierarchical structure has no random effects for site ($\varepsilon_{\lambda ,i}$, $\varepsilon_{p\left(\text{site}\right),i}$) or survey ($\varepsilon_{p\left(\text{survey}\right),i,j}$) on abundance and detection, nor their associated hyperparameters (Kery & Schaub, 2012, ch. 13.5.1).

## APPENDIX 2

### POSTERIORS

**Table A1:** Single‐species, multiseason occupancy model including year‐specific parameters and their hyperparameters (more hierarchical): posterior mean, *SD*, and Geweke *Z*‐score for chain convergence

	p=.27	block_AFSS			block_RW			jags_like_nimble			nimble		
		Mean	*SD*	*Z*	Mean	*SD*	*Z*	Mean	*SD*	*Z*	Mean	*SD*	*Z*
Latent state integration	psi1	0.27	0.06	0.63	0.27	0.06	0.73	0.27	0.06	0.47	0.27	0.06	0.48
	mu.p	−1.08	0.07	−1.02	−1.08	0.07	−0.89	−1.08	0.07	−0.38	−1.08	0.07	1.64
	sigma.p	0.13	0.08	−1.09	0.13	0.08	0.49	0.13	0.08	0.29	0.13	0.08	−1.79
	mu.phi	−1.08	0.19	1.02	−1.08	0.19	0.62	−1.08	0.19	1.6	−1.08	0.19	−1.28
	mu.gamma	−0.17	0.12	0.95	−0.17	0.12	−0.45	−0.17	0.12	0.25	−0.17	0.12	−1.07
	sigma.phi	0.38	0.23	−1.14	0.38	0.24	1.09	0.38	0.24	−0.2	0.38	0.23	−1.38
	sigma.gamma	0.13	0.1	−0.89	0.13	0.1	−1.5	0.13	0.1	1.79	0.13	0.1	−0.57

	p=.27	block_AFSS			block_RW			jags_like_nimble			jags			nimble		
		Mean	*SD*	*Z*	Mean	*SD*	*Z*	Mean	*SD*	*Z*	Mean	*SD*	*Z*	Mean	*SD*	*Z*
Latent states sampled	psi1	0.27	0.06	−0.69	0.27	0.06	1.26	0.27	0.06	−0.69	0.27	0.06	2.64	0.27	0.06	2.33
	mu.p	−1.08	0.07	−0.87	−1.08	0.07	0.72	−1.08	0.07	−0.74	−1.08	0.07	−2.43	−1.08	0.07	−0.26
	sigma.p	0.13	0.08	−1.15	0.13	0.08	0.47	0.13	0.08	−0.64	0.13	0.08	1.03	0.13	0.08	−0.76
	mu.phi	−1.08	0.19	1.77	−1.08	0.19	−0.51	−1.08	0.19	0.19	−1.08	0.19	1.12	−1.08	0.19	−1.42
	mu.gamma	−0.17	0.12	1.72	−0.17	0.12	0.82	−0.17	0.12	0.78	−0.17	0.12	2.92	−0.17	0.12	−0.04
	sigma.phi	0.38	0.24	−2.23	0.38	0.24	0.62	0.38	0.24	0.15	0.39	0.24	0.39	0.39	0.24	2.49
	sigma.gamma	0.13	0.1	0.2	0.12	0.1	0.64	0.13	0.1	1.45	0.13	0.1	−1.51	0.13	0.1	−0.56

	p=.73	block_AFSS			block_RW			jags_like_nimble			nimble		
		Mean	*SD*	*Z*	Mean	*SD*	*Z*	Mean	*SD*	*Z*	Mean	*SD*	*Z*
Latent state integration	psi1	0.26	0.08	−0.74	0.26	0.08	0.11	0.26	0.08	0.81	0.26	0.08	0.13
	mu.p	0.93	0.11	0.15	0.93	0.11	−1.38	0.93	0.11	−0.02	0.93	0.11	−0.81
	sigma.p	0.16	0.12	−0.74	0.16	0.12	−0.96	0.16	0.12	0.23	0.16	0.12	2.04
	mu.phi	−1.21	0.23	2.47	−1.21	0.23	−0.37	−1.21	0.23	1.05	−1.21	0.23	−0.21
	mu.gamma	−0.21	0.17	−1.65	−0.21	0.17	0.16	−0.21	0.17	0.83	−0.22	0.17	1.38
	sigma.phi	0.23	0.19	0.09	0.24	0.19	0.64	0.24	0.19	−1.52	0.24	0.18	−1.24
	sigma.gamma	0.3	0.2	−1.66	0.3	0.2	1.22	0.3	0.21	2.23	0.3	0.2	−1.08

	p=0.73	block_AFSS			block_RW			jags_like_nimble			jags			nimble		
		Mean	*SD*	*Z*	Mean	*SD*	*Z*	Mean	*SD*	*Z*	Mean	*SD*	*Z*	Mean	*SD*	*Z*
Latent states sampled	psi1	0.26	0.08	0.97	0.26	0.08	−0.74	0.26	0.08	−0.46	0.26	0.08	−0.37	0.26	0.08	0.37
	mu.p	0.93	0.11	−0.86	0.93	0.11	−0.55	0.93	0.11	0.48	0.93	0.11	1	0.94	0.11	−1.5
	sigma.p	0.16	0.12	−0.25	0.16	0.12	0.44	0.16	0.12	−1.79	0.16	0.12	0.13	0.16	0.12	−1.76
	mu.phi	−1.21	0.23	0.99	−1.21	0.23	−0.72	−1.21	0.23	−0.48	−1.21	0.23	−1.55	−1.21	0.23	−1.46
	mu.gamma	−0.22	0.17	−1.92	−0.21	0.17	−0.88	−0.22	0.17	0.99	−0.22	0.17	0.69	−0.22	0.17	3.59
	sigma.phi	0.24	0.19	1.38	0.23	0.19	−1.84	0.24	0.19	1.77	0.24	0.19	1.09	0.24	0.19	−1.03
	sigma.gamma	0.3	0.21	1.19	0.3	0.21	−1.4	0.31	0.2	0.02	0.31	0.2	−1.95	0.32	0.2	−1.42

The Geweke *Z*‐score is a standard *Z*‐score with a standard normal distribution. If the Geweke *Z*‐score is >1.96 or <−1.96, this indicates possible issues with chain convergence. We combined this metric with visual inspection of the chains to assess convergence.

**Table A2:** Single‐species, multiseason occupancy model excluding year‐specific parameters and their hyperparameters (less hierarchical): posterior Mean, *SD*, and Geweke *Z*‐score for chain convergence

	p=.27	block_AFSS			block_RW			jags_like_nimble			nimble		
		Mean	*SD*	*Z*	Mean	*SD*	*Z*	Mean	*SD*	*Z*	Mean	*SD*	*Z*
Latent state integration	psi1	0.26	0.05	−2.01	0.26	0.05	−0.82	0.26	0.05	0.1	0.26	0.05	0.68
	mu.p	−1.07	0.06	1.45	−1.07	0.06	1.59	−1.07	0.06	1.57	−1.07	0.06	0.25
	mu.phi	−1.06	0.14	0.02	−1.06	0.14	0	−1.06	0.14	1.06	−1.06	0.14	−1.17
	mu.gamma	−0.18	0.11	−0.51	−0.18	0.11	−0.54	−0.18	0.11	−1.82	−0.18	0.11	0.89

	p=.27	block_AFSS			block_RW			jags_like_nimble			jags			nimble		
		Mean	*SD*	*Z*	Mean	*SD*	*Z*	Mean	*SD*	*Z*	Mean	*SD*	*Z*	Mean	*SD*	*Z*
Latent states sampled	psi1	0.26	0.05	0.39	0.26	0.05	−0.99	0.26	0.05	0.77	0.26	0.05	1.02	0.26	0.05	1.14
	mu.p	−1.07	0.06	−0.37	−1.07	0.06	−0.36	−1.07	0.06	−1.01	−1.07	0.06	0.54	−1.07	0.06	0.62
	mu.phi	−1.06	0.14	−0.46	−1.06	0.14	−0.78	−1.06	0.14	0.35	−1.06	0.14	−0.66	−1.06	0.14	0.11
	mu.gamma	−0.18	0.11	0.64	−0.18	0.11	1.3	−0.18	0.11	0.48	−0.18	0.11	−0.33	−0.18	0.11	−1.05

	p=.73	block_AFSS			block_RW			jags_like_nimble			nimble		
		Mean	*SD*	*Z*	Mean	*SD*	*Z*	Mean	*SD*	*Z*	Mean	*SD*	*Z*
Latent state integration	psi1	0.26	0.08	1.72	0.26	0.08	−0.34	0.26	0.08	−0.33	0.26	0.08	0.36
	mu.p	0.93	0.1	−1.03	0.93	0.1	0.67	0.93	0.1	0.35	0.93	0.1	−0.82
	mu.phi	−1.2	0.21	−0.15	−1.2	0.21	−1.73	−1.2	0.21	1.37	−1.2	0.21	1.02
	mu.gamma	−0.21	0.14	−0.45	−0.21	0.14	−0.36	−0.21	0.14	0.39	−0.21	0.14	3

	p=.73	block_AFSS			block_RW			jags_like_nimble			jags			nimble		
		Mean	*SD*	*Z*	Mean	*SD*	*Z*	Mean	*SD*	*Z*	Mean	*SD*	*Z*	Mean	*SD*	*Z*
Latent states sampled	psi1	0.26	0.08	−0.05	0.26	0.08	−0.18	0.26	0.08	0.06	0.26	0.08	2.42	0.26	0.08	−0.12
	mu.p	0.93	0.1	0.3	0.93	0.1	0.56	0.93	0.1	−0.18	0.93	0.1	0.31	0.93	0.1	0.34
	mu.phi	−1.2	0.21	−2.11	−1.2	0.22	−0.45	−1.2	0.21	−0.73	−1.2	0.21	−0.15	−1.2	0.21	2.36
	mu.gamma	−0.21	0.14	0.15	−0.21	0.14	1.27	−0.21	0.14	0.76	−0.21	0.14	−0.15	−0.21	0.14	−1.37

**Table A3:** Multispecies, single‐season occupancy model including species‐specific parameters and their hyperparameters (more hierarchical): posterior mean, *SD*, and Geweke *Z*‐score for chain convergence

	block_AFSS			block_RW			jags_like_nimble			nimble		
	Mean	*SD*	*Z*	Mean	*SD*	*Z*	Mean	*SD*	*Z*	Mean	*SD*	*Z*
Latent state integration												
cato.occ.mean	0.43	0.15	0.38	0.43	0.15	1.44	0.43	0.15	−0.33	0.43	0.15	0.51
sigma.ucato	3.67	0.87	0.71	3.62	0.9	−1.08	3.68	0.87	−1.35	3.66	0.85	0.7
fcw.occ.mean	0.52	0.12	1.1	0.52	0.12	−3.23	0.52	0.12	−1.14	0.52	0.12	−0.32
sigma.ufcw	2.99	0.57	−0.44	2.97	0.56	0.53	2.99	0.57	−1.51	3	0.58	1.09
cato.det.mean	0.18	0.05	−0.04	0.18	0.05	−0.7	0.18	0.04	−0.43	0.18	0.04	−0.81
sigma.vcato	1.48	0.28	0.46	1.49	0.28	0.44	1.47	0.27	0.88	1.48	0.27	0.35
fcw.det.mean	0.24	0.05	−1.7	0.24	0.05	1.85	0.24	0.05	0.87	0.24	0.05	1.17
sigma.vfcw	1.32	0.23	1.63	1.33	0.23	−1.9	1.32	0.23	−0.27	1.32	0.23	−2.89
mu.a1	0.51	0.17	−0.21	0.49	0.17	−0.11	0.51	0.17	−1.55	0.51	0.17	1.42
sigma.a1	0.73	0.18	−0.04	0.72	0.18	−1.3	0.73	0.18	−2.1	0.73	0.18	−0.69
mu.a2	0.03	0.12	0.35	0.02	0.12	1.09	0.03	0.12	0.58	0.03	0.12	−2.47
sigma.a2	0.25	0.18	−1.2	0.25	0.18	−0.57	0.26	0.18	−0.54	0.25	0.18	−0.49
mu.a3	−0.16	0.11	0.63	−0.17	0.1	2.48	−0.16	0.11	−0.27	−0.16	0.11	−3.12
sigma.a3	0.17	0.13	1.03	0.14	0.12	−0.36	0.17	0.13	−0.68	0.18	0.13	1.71
mu.a4	0.13	0.09	0.08	0.13	0.09	−3.66	0.13	0.09	0.14	0.13	0.09	0.73
sigma.a4	0.28	0.15	−2.63	0.27	0.16	−0.28	0.28	0.15	1.69	0.28	0.15	4.18
mu.b1	−0.13	0.05	1.27	−0.13	0.05	0.94	−0.13	0.05	−0.24	−0.13	0.05	0.35
sigma.b1	0.21	0.06	−0.14	0.21	0.06	−0.18	0.21	0.06	−0.39	0.21	0.06	0.05
mu.b2	0.08	0.03	0.14	0.09	0.03	0.05	0.09	0.03	0.69	0.08	0.03	3
sigma.b2	0.04	0.03	−0.13	0.04	0.03	1.24	0.04	0.03	−0.4	0.04	0.03	−0.71

	block_AFSS			block_RW			jags_like_nimble			jags			nimble		
	Mean	*SD*	*Z*	Mean	*SD*	*Z*	Mean	*SD*	*Z*	Mean	*SD*	*Z*	Mean	*SD*	*Z*
Latent states sampled															
cato.occ.mean	0.42	0.15	0.89	0.43	0.15	−3.37	0.43	0.15	0.42	0.43	0.15	0.68	0.43	0.14	2.07
sigma.ucato	3.68	0.86	−0.01	3.59	0.84	−1.11	3.68	0.86	1.82	3.68	0.88	−0.04	3.66	0.87	1.28
fcw.occ.mean	0.52	0.12	0.52	0.52	0.12	0.75	0.52	0.12	−0.43	0.52	0.12	0.96	0.52	0.12	−0.22
sigma.ufcw	3	0.58	0.37	2.96	0.58	0.36	2.99	0.58	−0.45	2.99	0.57	−2.25	2.98	0.57	0.28
cato.det.mean	0.18	0.04	−1.77	0.18	0.05	2.13	0.18	0.04	0.19	0.18	0.04	−0.52	0.18	0.04	−1.89
sigma.vcato	1.47	0.27	1.21	1.5	0.3	−2.15	1.47	0.27	−0.17	1.48	0.27	0.65	1.47	0.26	1.66
fcw.det.mean	0.24	0.05	−0.82	0.23	0.05	−0.66	0.24	0.05	−0.35	0.24	0.05	−1.26	0.24	0.05	0.23
sigma.vfcw	1.32	0.23	0.64	1.35	0.24	1.27	1.33	0.23	−0.25	1.32	0.23	1.77	1.32	0.22	−0.33
mu.a1	0.51	0.17	0.61	0.49	0.18	1.91	0.51	0.17	2.03	0.51	0.17	1.38	0.5	0.17	1.74
sigma.a1	0.73	0.18	−1.06	0.73	0.17	−0.09	0.73	0.18	−0.55	0.73	0.18	0.79	0.73	0.18	0.31
mu.a2	0.02	0.12	0.26	0.03	0.13	0.87	0.03	0.12	0.33	0.03	0.12	0.65	0.03	0.12	0.04
sigma.a2	0.23	0.18	−0.44	0.26	0.17	0.3	0.25	0.18	−0.69	0.25	0.18	0.4	0.26	0.19	−0.18
mu.a3	−0.16	0.11	−0.32	−0.16	0.11	0.93	−0.16	0.11	1.83	−0.16	0.11	2.74	−0.16	0.11	−1.09
sigma.a3	0.17	0.13	−3.17	0.18	0.12	−0.45	0.17	0.13	1.39	0.17	0.12	−1.82	0.18	0.12	−0.06
mu.a4	0.13	0.09	0.87	0.13	0.09	0.67	0.13	0.09	−1.08	0.13	0.09	−1.2	0.13	0.09	0.71
sigma.a4	0.28	0.15	1.52	0.29	0.14	−0.26	0.27	0.15	−0.42	0.28	0.15	0.39	0.29	0.14	−0.37
mu.b1	−0.13	0.05	−0.28	−0.13	0.05	−0.28	−0.13	0.05	−0.37	−0.13	0.05	−0.03	−0.13	0.05	1.55
sigma.b1	0.21	0.06	1.31	0.21	0.06	−0.41	0.21	0.06	0.24	0.21	0.06	0.88	0.21	0.06	0.95
mu.b2	0.09	0.03	2.91	0.09	0.03	−1.18	0.09	0.03	0.61	0.09	0.03	−1.4	0.08	0.03	−0.54
sigma.b2	0.04	0.03	0.18	0.04	0.03	1.1	0.04	0.03	0.15	0.04	0.03	−1.24	0.04	0.03	−0.07

**Table A4:** Multispecies, single‐season occupancy model excluding species‐specific parameters and their hyperparameters (less hierarchical): posterior mean, *SD*, and Geweke *Z*‐score for chain convergence

	block_AFSS			block_RW			jags_like_nimble			nimble		
	Mean	*SD*	*Z*	Mean	*SD*	*Z*	Mean	*SD*	*Z*	Mean	*SD*	*Z*
Latent state integration												
a1	0.14	0.05	−0.37	0.14	0.05	1.29	0.14	0.05	0.74	0.14	0.05	−1.27
a2	0	0.04	−0.16	0	0.04	−1.73	0	0.04	−0.04	0	0.04	0.91
a3	−0.06	0.04	−2.54	−0.06	0.04	0.62	−0.06	0.04	0.29	−0.06	0.04	−0.17
a4	0.06	0.03	−0.43	0.06	0.03	−0.3	0.06	0.03	0.39	0.06	0.03	−0.17
b1	−0.1	0.03	−0.12	−0.1	0.03	0.74	−0.1	0.03	−0.75	−0.1	0.03	−0.63
b2	0.09	0.03	1.39	0.09	0.03	−1.15	0.09	0.03	0.48	0.09	0.03	−0.17
u.cato	−0.98	0.07	−0.27	−0.98	0.07	1.6	−0.98	0.07	−1.01	−0.98	0.07	−0.19
u.fcw	−0.66	0.08	−0.08	−0.66	0.08	1.84	−0.66	0.08	−0.24	−0.66	0.08	−0.55
v.cato	−0.34	0.07	1.01	−0.34	0.07	0.36	−0.34	0.07	0.72	−0.34	0.07	0.33
v.fcw	−0.22	0.07	0.33	−0.22	0.07	−0.84	−0.22	0.07	−0.54	−0.22	0.07	0.04

	block_AFSS			block_RW			jags_like_nimble			jags			nimble		
	Mean	*SD*	*Z*	Mean	*SD*	*Z*	Mean	*SD*	*Z*	Mean	*SD*	*Z*	Mean	*SD*	*Z*
Latent states sampled															
a1	0.14	0.05	0.15	0.14	0.05	−0.28	0.14	0.05	−0.23	0.14	0.05	−0.41	0.14	0.05	0.57
a2	0	0.04	−1	0	0.04	0.52	0	0.04	−0.87	0	0.04	0.17	0	0.04	−0.78
a3	−0.06	0.04	−0.27	−0.06	0.04	1.01	−0.06	0.04	0.32	−0.06	0.04	0.44	−0.06	0.04	0.25
a4	0.06	0.03	0.87	0.06	0.03	0.48	0.06	0.03	−1.04	0.06	0.03	−1.3	0.06	0.03	−1.01
b1	−0.1	0.03	1.13	−0.1	0.03	0.61	−0.1	0.03	0.16	−0.1	0.03	0.26	−0.1	0.03	0.01
b2	0.09	0.03	−1.15	0.09	0.03	−1.7	0.09	0.03	0.44	0.09	0.03	1.09	0.09	0.03	−0.99
u.cato	−0.98	0.07	0.99	−0.98	0.07	1.11	−0.98	0.07	0.82	−0.98	0.07	0.09	−0.98	0.07	0.67
u.fcw	−0.66	0.08	−0.49	−0.67	0.08	−0.2	−0.66	0.08	1.41	−0.66	0.08	0.41	−0.67	0.08	1.5
v.cato	−0.34	0.07	0.31	−0.34	0.07	−0.38	−0.34	0.07	0.34	−0.34	0.07	0.07	−0.34	0.07	1.44
v.fcw	−0.22	0.07	0.36	−0.22	0.07	0.63	−0.22	0.07	−1.14	−0.22	0.07	−1.72	−0.22	0.07	0.42

**Table A5:** Multispecies, multiseason occupancy model including species‐specific parameters and their hyperparameters (more hierarchical): posterior mean, *SD*, and Geweke *Z*‐score for chain convergence

	block_AFSS			block_RW			jags_like_nimble			nimble		
	Mean	*SD*	*Z*	Mean	*SD*	*Z*	Mean	*SD*	*Z*	Mean	*SD*	*Z*
Latent state integration												
mu.p.0	−2.32	0.18	−1.55	−2.32	0.18	0.61	−2.32	0.18	−5.31	−2.33	0.18	−1.66
mu.p.day.1	0.29	0.15	−0.6	0.29	0.15	−0.77	0.29	0.15	1.4	0.29	0.15	−0.41
mu.p.day.2	−0.24	0.07	−0.4	−0.24	0.07	−0.54	−0.24	0.07	−0.89	−0.24	0.07	−1.57
sigma.p.0	0.99	0.14	2.13	0.99	0.14	−0.53	0.99	0.14	3.89	0.99	0.14	1.72
sigma.p.day.1	0.98	0.15	0.2	0.98	0.15	0.93	0.98	0.15	0.01	0.98	0.15	1.29
sigma.p.day.2	0.3	0.09	0.7	0.29	0.08	0.07	0.3	0.09	−0.7	0.29	0.09	−0.11
mu.phi.0	0.36	0.27	1.37	0.38	0.24	0.06	0.37	0.27	2.04	0.37	0.27	−0.33
mu.gam.0	−2.21	0.16	0.46	−2.21	0.16	0.73	−2.21	0.16	1.18	−2.21	0.16	2.01
sigma.phi.0	0.41	0.22	−1.05	0.41	0.22	0.56	0.41	0.22	1.35	0.42	0.22	2.63
sigma.gam.0	0.66	0.13	−1.82	0.66	0.13	0.68	0.66	0.13	0.67	0.66	0.14	0
mu.phi.hr.area	−0.47	0.26	0.22	−0.42	0.26	0.04	−0.45	0.26	−3.17	−0.44	0.26	−0.64
mu.gam.hr.area	0.4	0.17	0.42	0.38	0.16	1.23	0.39	0.16	4.37	0.39	0.17	0.58
sigma.phi.hr.area	0.23	0.16	−0.44	0.25	0.16	−1.64	0.23	0.17	−1.77	0.25	0.16	−1.94
sigma.gam.hr.area	0.13	0.1	−1.27	0.14	0.1	0.93	0.14	0.1	2.95	0.14	0.1	0.61
mu.phi.nat.area	0.27	0.26	0.88	0.23	0.24	3.45	0.27	0.24	3.78	0.25	0.27	0.8
mu.gam.nat.area	−0.25	0.17	−0.84	−0.24	0.15	−3.48	−0.26	0.16	−3.44	−0.25	0.18	−0.46
sigma.phi.nat.area	0.28	0.18	−0.59	0.28	0.17	0.68	0.26	0.18	−0.26	0.27	0.18	1.4
sigma.gam.nat.area	0.11	0.08	0.37	0.11	0.08	−3.61	0.11	0.08	0.4	0.12	0.08	0.44
mu.phi.fra	0.65	0.21	−0.62	0.62	0.21	1.6	0.63	0.21	1.24	0.63	0.21	2.8
mu.gam.fra	0.31	0.11	−0.51	0.32	0.11	−3.56	0.32	0.1	−1.84	0.33	0.11	−2.22
sigma.phi.fra	0.46	0.22	−0.08	0.48	0.22	0.29	0.44	0.23	0.89	0.47	0.22	2.17
sigma.gam.fra	0.14	0.1	0.72	0.13	0.1	−4.12	0.14	0.1	1.53	0.15	0.1	1.02
phi.k	0.85	0.26	−1.26	0.83	0.26	−1.27	0.84	0.27	−2.8	0.84	0.26	−1.06
gam.k	0.33	0.16	−1.03	0.34	0.15	−0.52	0.34	0.16	−0.24	0.34	0.16	0.03
phi.B	−0.06	0.18	−0.6	−0.06	0.19	−2.54	−0.06	0.18	−0.45	−0.06	0.18	−2.28
gam.B	−0.37	0.15	0.97	−0.37	0.15	1.03	−0.38	0.15	−1.92	−0.38	0.15	0.83
phi.hr.area.fra	−0.31	0.27	−0.41	−0.35	0.25	−0.97	−0.31	0.27	0.97	−0.33	0.27	2.12
gam.hr.area.fra	0.33	0.15	−0.41	0.35	0.13	0.4	0.33	0.15	−1.01	0.34	0.15	−3.43
phi.nat.area.fra	0.06	0.23	−0.19	0.07	0.23	1.2	0.05	0.23	−2.74	0.06	0.23	−2.32
gam.nat.area.fra	−0.31	0.14	0.46	−0.31	0.14	−3.16	−0.3	0.15	−0.6	−0.31	0.15	1.79
phi.hr.area.k	0.7	0.25	0.1	0.67	0.24	−0.4	0.69	0.25	4.39	0.68	0.25	0.38
gam.hr.area.k	−0.25	0.17	−1.52	−0.24	0.16	−1.72	−0.25	0.17	−6.99	−0.25	0.17	−1.05
phi.nat.area.k	−0.09	0.26	−2.01	−0.07	0.25	−3.96	−0.09	0.25	−6.37	−0.08	0.26	−0.39
gam.nat.area.k	−0.06	0.18	2.27	−0.07	0.17	4.61	−0.06	0.18	6.69	−0.06	0.17	0.79
phi.hr.area.B	0.03	0.21	−0.38	0.02	0.21	−0.51	0.03	0.21	2.11	0.02	0.21	−0.87
gam.hr.area.B	0.01	0.15	0.49	0.02	0.15	1.98	0.01	0.15	−2.31	0.01	0.15	0.22
phi.nat.area.B	0.36	0.22	0.45	0.37	0.22	0.78	0.36	0.22	−1.4	0.36	0.21	0.68
gam.nat.area.B	−0.26	0.15	−0.39	−0.26	0.15	−0.16	−0.26	0.15	1.76	−0.26	0.15	0.02

	block_AFSS			block_RW			jags_like_nimble			jags			nimble		
	Mean	*SD*	*Z*	Mean	*SD*	*Z*	Mean	*SD*	*Z*	Mean	*SD*	*Z*	Mean	*SD*	*Z*
Latent states sampled															
mu.p.0	−2.33	0.18	0.25	−2.34	0.18	3.45	−2.32	0.18	1.82	−2.32	0.18	−2.52	−2.32	0.18	1.78
mu.p.day.1	0.29	0.15	−0.61	0.29	0.15	−0.37	0.29	0.15	1.09	0.29	0.15	0.45	0.29	0.15	0.49
mu.p.day.2	−0.24	0.07	0.45	−0.24	0.07	−0.37	−0.24	0.07	−0.78	−0.24	0.07	0.07	−0.24	0.07	0.75
sigma.p.0	1	0.14	−0.1	1	0.14	−4.01	0.99	0.14	−0.37	0.99	0.14	1.91	0.99	0.14	−1.01
sigma.p.day.1	0.98	0.15	−1.83	0.98	0.15	−1.18	0.98	0.15	0.21	0.97	0.15	−0.34	0.98	0.15	−0.41
sigma.p.day.2	0.3	0.09	2.72	0.3	0.09	1.23	0.29	0.09	0.95	0.3	0.09	0.24	0.3	0.09	0.29
mu.phi.0	0.39	0.3	−1.49	0.41	0.27	0.53	0.37	0.27	0.95	0.37	0.27	1.81	0.4	0.28	0.61
mu.gam.0	−2.21	0.16	2.36	−2.22	0.16	−1.45	−2.21	0.16	−2.29	−2.21	0.16	−0.03	−2.22	0.16	−3.06
sigma.phi.0	0.41	0.22	1.69	0.39	0.23	−0.82	0.41	0.22	1.16	0.4	0.22	0.71	0.42	0.22	1.92
sigma.gam.0	0.66	0.13	−2.17	0.65	0.13	−0.23	0.66	0.13	−2.21	0.66	0.13	0.61	0.66	0.13	0.21
mu.phi.hr.area	−0.49	0.3	−0.26	−0.48	0.28	2.14	−0.45	0.26	1.31	−0.42	0.26	−0.35	−0.4	0.27	2.38
mu.gam.hr.area	0.43	0.19	−0.8	0.42	0.17	−1.31	0.4	0.16	−0.97	0.38	0.17	0.87	0.37	0.17	−2.82
sigma.phi.hr.area	0.22	0.16	0.16	0.22	0.16	0.25	0.22	0.16	−0.53	0.22	0.16	−0.79	0.24	0.16	0.91
sigma.gam.hr.area	0.14	0.1	0.73	0.13	0.09	−1.72	0.14	0.1	0.12	0.14	0.1	0.44	0.13	0.09	−0.02
mu.phi.nat.area	0.33	0.28	−0.84	0.29	0.23	−3.21	0.28	0.25	1.16	0.24	0.26	0.45	0.25	0.26	−1.87
mu.gam.nat.area	−0.29	0.19	1.7	−0.27	0.15	2.04	−0.26	0.17	−0.43	−0.24	0.17	−0.6	−0.24	0.18	2.49
sigma.phi.nat.area	0.28	0.19	−0.06	0.26	0.18	−0.86	0.27	0.18	0.31	0.28	0.19	−1.64	0.28	0.18	−0.81
sigma.gam.nat.area	0.11	0.08	−0.15	0.1	0.08	−1.27	0.11	0.08	0.02	0.11	0.09	0.59	0.11	0.08	1.11
mu.phi.fra	0.63	0.21	1.69	0.61	0.2	−1.13	0.64	0.21	−1.77	0.63	0.22	−0.6	0.62	0.21	−2.93
mu.gam.fra	0.32	0.11	−1.65	0.33	0.1	0.81	0.32	0.11	0.74	0.32	0.11	1.16	0.33	0.11	2.42
sigma.phi.fra	0.46	0.22	−3.51	0.43	0.21	1.74	0.46	0.23	1.67	0.45	0.23	0.69	0.47	0.22	−2.4
sigma.gam.fra	0.14	0.1	−0.95	0.15	0.1	0.07	0.13	0.1	1.94	0.14	0.1	1.53	0.15	0.1	0.49
phi.k	0.81	0.29	1.01	0.83	0.27	−0.51	0.85	0.26	−2.08	0.85	0.26	−1.83	0.81	0.28	0.25
gam.k	0.34	0.16	−1.39	0.33	0.15	0.37	0.33	0.16	2.9	0.33	0.16	0.72	0.34	0.16	−0.1
phi.B	−0.07	0.18	0.83	−0.06	0.18	−0.26	−0.05	0.19	−1.92	−0.06	0.18	0.13	−0.06	0.19	1.08
gam.B	−0.37	0.15	−0.61	−0.39	0.15	1.36	−0.38	0.15	2.35	−0.38	0.15	0.87	−0.38	0.15	0.69
phi.hr.area.fra	−0.32	0.26	3.22	−0.3	0.29	−4.16	−0.33	0.27	−0.89	−0.32	0.27	−0.15	−0.33	0.26	2.61
gam.hr.area.fra	0.34	0.14	−2.71	0.33	0.16	3.46	0.34	0.15	0.54	0.34	0.15	−0.3	0.34	0.14	−1.72
phi.nat.area.fra	0.05	0.22	−1.63	0.05	0.24	4.31	0.06	0.23	−0.15	0.06	0.23	−0.39	0.04	0.23	−2.7
gam.nat.area.fra	−0.31	0.14	1.64	−0.3	0.15	−2.58	−0.31	0.14	−0.58	−0.31	0.15	1.13	−0.3	0.14	2.84
phi.hr.area.k	0.72	0.26	−0.15	0.7	0.25	−4.72	0.69	0.24	−1.8	0.68	0.24	0.5	0.67	0.25	−2.98
gam.hr.area.k	−0.29	0.18	2.23	−0.27	0.17	3.69	−0.25	0.16	0.19	−0.25	0.16	−1.64	−0.25	0.17	3.73
phi.nat.area.k	−0.14	0.27	1.62	−0.1	0.25	5.23	−0.09	0.25	−1.13	−0.08	0.26	−1	−0.09	0.26	2.5
gam.nat.area.k	−0.02	0.19	−3.1	−0.05	0.18	−5.14	−0.06	0.17	0.91	−0.06	0.18	1.17	−0.05	0.18	−3.21
phi.hr.area.B	0.05	0.21	−0.04	0.03	0.2	0.92	0.02	0.21	−0.46	0.03	0.2	−0.33	0.02	0.21	−1.84
gam.hr.area.B	0	0.15	−0.31	0.01	0.15	−0.89	0.01	0.15	0.38	0.01	0.15	0.22	0.01	0.15	1.78
phi.nat.area.B	0.35	0.22	1.41	0.36	0.21	−1.44	0.36	0.21	−0.81	0.36	0.22	−0.77	0.36	0.23	1.29
gam.nat.area.B	−0.25	0.16	−1.12	−0.26	0.15	0.91	−0.26	0.15	1.39	−0.26	0.16	0.25	−0.26	0.16	−1.34

**Table A6:** Multispecies, multiseason occupancy model excluding species‐specific parameters and their hyperparameters (less hierarchical) posterior mean, *SD*, and Geweke *Z*‐score for chain convergence

	block_AFSS			block_RW			jags_like_nimble			nimble		
	Mean	*SD*	*Z*	Mean	*SD*	*Z*	Mean	*SD*	*Z*	Mean	*SD*	*Z*
Latent state integration												
p.0	−1.23	0.05	−0.53	−1.23	0.05	1.61	−1.23	0.05	1.47	−1.23	0.05	0.85
p.day.1	0.37	0.03	2.82	0.37	0.03	0.22	0.37	0.03	0.26	0.37	0.03	1.35
p.day.2	−0.09	0.03	−1.09	−0.09	0.03	0.07	−0.09	0.03	−1.2	−0.09	0.03	0.67
phi.0	−0.34	0.2	0.14	−0.35	0.2	0.59	−0.35	0.2	−1.48	−0.35	0.2	0.1
gam.0	−2.55	0.07	1.69	−2.55	0.07	−0.39	−2.55	0.07	0.48	−2.55	0.07	1.53
phi.hr.area	−0.58	0.27	1.61	−0.58	0.27	1.77	−0.58	0.27	−0.63	−0.58	0.27	−1.6
gam.hr.area	0.29	0.12	−1.25	0.29	0.12	−2.13	0.29	0.12	0.03	0.28	0.12	1.21
phi.nat.area	0.23	0.22	−1.49	0.24	0.22	−1.26	0.24	0.22	0.46	0.23	0.22	1.39
gam.nat.area	−0.19	0.11	1.54	−0.19	0.11	1.05	−0.19	0.11	0.18	−0.19	0.11	−1.19
phi.fra	0.53	0.16	−0.64	0.53	0.16	−1.38	0.53	0.16	2.33	0.53	0.15	0.46
gam.fra	0.25	0.08	1.31	0.25	0.08	0.66	0.25	0.08	−2.28	0.25	0.08	−0.51
phi.k	1.55	0.17	−0.23	1.56	0.17	−0.68	1.56	0.17	0.85	1.56	0.17	−1.18
gam.k	0.28	0.08	−1.77	0.28	0.08	0.24	0.28	0.08	0.2	0.28	0.08	1.17
phi.B	−0.2	0.12	−1.23	−0.21	0.12	2.4	−0.2	0.12	−0.68	−0.21	0.12	−0.11
gam.B	−0.18	0.07	1.37	−0.18	0.07	−1.99	−0.18	0.07	−0.63	−0.18	0.07	0.45
phi.hr.area.fra	0.17	0.26	1.01	0.17	0.25	−0.76	0.17	0.26	−0.09	0.19	0.26	1.1
gam.hr.area.fra	0.06	0.18	−1.18	0.06	0.17	0.03	0.06	0.17	0.81	0.05	0.17	−1.7
phi.nat.area.fra	0.07	0.21	−1.29	0.07	0.2	0.2	0.07	0.2	0.7	0.06	0.2	−1.41
gam.nat.area.fra	−0.24	0.14	1.39	−0.24	0.14	0.6	−0.24	0.14	−0.97	−0.23	0.14	2.13
phi.hr.area.k	0.51	0.27	−1.33	0.51	0.27	−0.56	0.51	0.27	−0.09	0.51	0.27	2.1
gam.hr.area.k	−0.06	0.13	0.59	−0.06	0.13	−0.83	−0.06	0.13	0.78	−0.06	0.13	−2.74
phi.nat.area.k	0.01	0.21	0.95	0.01	0.22	−0.14	0	0.22	0.13	0	0.21	−2.01
gam.nat.area.k	−0.11	0.12	−0.45	−0.11	0.12	1.39	−0.11	0.12	−0.71	−0.11	0.12	2.79
phi.hr.area.B	0.12	0.2	0.58	0.13	0.21	−0.61	0.12	0.2	0.74	0.12	0.2	−3.03
gam.hr.area.B	0	0.13	−0.44	−0.01	0.13	0.49	0	0.13	−1.25	0	0.13	2.59
phi.nat.area.B	0.22	0.19	−0.33	0.21	0.19	0.17	0.22	0.19	−0.6	0.22	0.19	2.44
gam.nat.area.B	−0.14	0.12	0.29	−0.14	0.12	−0.06	−0.14	0.12	1.19	−0.14	0.12	−2.15

	block_AFSS			block_RW			jags_like_nimble			jags			nimble		
	Mean	*SD*	*Z*	Mean	*SD*	*Z*	Mean	*SD*	*Z*	Mean	*SD*	*Z*	Mean	*SD*	*Z*
Latent states sampled															
p.0	−1.23	0.05	0.57	−1.23	0.05	−0.44	−1.23	0.05	0.02	−1.23	0.05	−0.32	−1.23	0.05	0.38
p.day.1	0.37	0.03	−1.07	0.37	0.03	−2.11	0.37	0.03	0.95	0.37	0.03	0.29	0.37	0.03	1.05
p.day.2	−0.09	0.03	0.2	−0.09	0.03	0.28	−0.09	0.03	−0.13	−0.09	0.03	0.99	−0.09	0.03	−0.61
phi.0	−0.35	0.2	−0.8	−0.33	0.2	−1.46	−0.35	0.2	1.34	−0.34	0.2	0.41	−0.33	0.2	−0.24
gam.0	−2.55	0.07	1.04	−2.55	0.07	2.96	−2.55	0.07	−1.56	−2.55	0.07	−0.58	−2.55	0.08	−0.6
phi.hr.area	−0.59	0.27	1.13	−0.57	0.28	−0.46	−0.59	0.27	1.39	−0.58	0.28	−1.67	−0.59	0.28	−0.83
gam.hr.area	0.29	0.12	−1.72	0.28	0.12	1.56	0.29	0.12	−2.33	0.29	0.12	1.38	0.29	0.12	1.16
phi.nat.area	0.24	0.22	−0.9	0.23	0.22	0.41	0.24	0.22	−0.64	0.23	0.22	2	0.24	0.22	−0.03
gam.nat.area	−0.19	0.11	0.7	−0.18	0.11	−1.18	−0.19	0.11	1.25	−0.19	0.11	−1.66	−0.19	0.11	−0.31
phi.fra	0.54	0.16	−0.16	0.53	0.16	0.31	0.54	0.16	−1.02	0.54	0.16	−0.44	0.53	0.15	0.92
gam.fra	0.25	0.08	−1.37	0.25	0.08	−0.29	0.25	0.08	0.71	0.25	0.08	0.69	0.25	0.08	0.4
phi.k	1.56	0.17	0.7	1.54	0.17	1.82	1.55	0.17	−0.83	1.56	0.17	−0.95	1.55	0.17	−0.39
gam.k	0.29	0.08	−0.53	0.29	0.08	−1.13	0.29	0.08	−0.26	0.28	0.08	1.13	0.28	0.08	1.08
phi.B	−0.2	0.11	0.51	−0.2	0.11	−2.56	−0.21	0.12	1.77	−0.21	0.12	1.38	−0.2	0.12	−1
gam.B	−0.18	0.07	0.15	−0.18	0.07	2.86	−0.18	0.07	−2	−0.18	0.07	−1.34	−0.18	0.07	0.37
phi.hr.area.fra	0.17	0.26	−0.57	0.15	0.27	2.3	0.17	0.26	−0.97	0.18	0.26	−0.24	0.17	0.26	0.63
gam.hr.area.fra	0.06	0.17	0.89	0.07	0.18	−1.88	0.06	0.18	0.6	0.05	0.18	0.25	0.06	0.18	0.25
phi.nat.area.fra	0.07	0.2	0.78	0.08	0.21	−2.38	0.07	0.21	0.73	0.06	0.2	0.96	0.07	0.21	0.15
gam.nat.area.fra	−0.25	0.14	−0.85	−0.25	0.14	1.72	−0.24	0.14	−0.35	−0.24	0.14	−0.77	−0.24	0.14	−0.69
phi.hr.area.k	0.52	0.26	−0.5	0.51	0.27	−0.95	0.52	0.26	0.31	0.52	0.28	1.26	0.53	0.27	0.92
gam.hr.area.k	−0.07	0.13	0.71	−0.06	0.13	1.45	−0.07	0.13	−0.61	−0.07	0.13	−0.74	−0.07	0.13	−1.18
phi.nat.area.k	−0.01	0.21	0.35	0.01	0.21	1.23	0	0.21	−0.47	0	0.22	−1.24	−0.01	0.21	−0.92
gam.nat.area.k	−0.1	0.12	−0.3	−0.11	0.12	−1.55	−0.11	0.12	−0.03	−0.1	0.12	0.23	−0.1	0.12	1.51
phi.hr.area.B	0.12	0.2	1.76	0.13	0.2	−0.93	0.1	0.2	−0.91	0.13	0.2	−1.85	0.11	0.21	1.84
gam.hr.area.B	−0.01	0.12	−1.8	−0.01	0.13	1.31	0.01	0.13	1.17	−0.01	0.13	2.12	0	0.13	−1.86
phi.nat.area.B	0.21	0.19	−1.61	0.21	0.19	1.01	0.23	0.19	0.62	0.21	0.19	1.82	0.22	0.2	−2.59
gam.nat.area.B	−0.14	0.12	1.67	−0.14	0.12	−1.08	−0.15	0.12	−0.86	−0.14	0.12	−2.03	−0.15	0.13	2.39

**Table A7:** Zero‐inflated N‐mixture model including species‐specific parameters and their hyperparameters (more hierarchical) posterior mean, *SD*, and Geweke *Z*‐score for chain convergence

	block_AFSS			block_RW			jags_like_nimble			nimble		
	Mean	*SD*	*Z*	Mean	*SD*	*Z*	Mean	*SD*	*Z*	Mean	*SD*	*Z*
Latent state integration												
phi	0.97	0.02	2.52	0.97	0.02	0.69	0.97	0.02	1.11	0.97	0.02	−1.03
beta0	3.32	0.29	1.54	3.27	0.28	−0.09	3.28	0.31	2.02	3.26	0.25	1.17
beta[1]	−0.29	0.27	0.72	−0.32	0.26	−1.59	−0.31	0.28	1.97	−0.35	0.24	0.53
beta[2]	−0.03	0.2	0.21	−0.03	0.2	−1.79	−0.02	0.2	−0.48	−0.05	0.2	−0.48
beta[3]	0.11	0.11	0.01	0.11	0.11	−0.19	0.12	0.11	−0.31	0.11	0.11	−0.51
beta[4]	−0.27	0.09	−0.24	−0.26	0.09	0.39	−0.27	0.09	0.18	−0.27	0.09	−0.17
beta[5]	−0.84	0.73	0.9	−0.85	0.73	−0.62	−0.84	0.72	0.37	−0.85	0.71	−2.06
beta[6]	−0.05	0.11	−0.74	−0.06	0.11	−0.37	−0.05	0.11	−0.01	−0.05	0.11	−0.58
beta[7]	−0.16	0.1	−1.08	−0.16	0.1	0.85	−0.16	0.1	−0.71	−0.16	0.1	−0.5
sd.lam	0.36	0.09	−1.54	0.38	0.08	1.26	0.38	0.09	−2.62	0.38	0.08	−0.15
mean.p[1]	0.26	0.07	−1.2	0.28	0.07	0.1	0.28	0.08	−1.65	0.28	0.07	−0.46
mean.p[2]	0.2	0.06	−1.2	0.21	0.06	0.08	0.21	0.06	−1.82	0.22	0.06	−0.45
mean.p[3]	0.16	0.04	−1.3	0.17	0.04	0.06	0.17	0.05	−2.02	0.17	0.04	−0.4
alpha[1]	−1.6	0.35	−0.39	−1.57	0.34	1.48	−1.59	0.36	−1.49	−1.53	0.33	0.7
alpha[2]	−0.54	0.31	1.3	−0.56	0.31	1.53	−0.56	0.31	1.37	−0.53	0.31	0.99
alpha[3]	−0.1	0.12	1.05	−0.1	0.13	0.01	−0.1	0.12	0.4	−0.1	0.12	−1.61
alpha[4]	0.07	0.07	1.08	0.07	0.07	1.9	0.06	0.07	−0.34	0.07	0.07	0.22
alpha[5]	−0.01	0.12	0.08	−0.01	0.12	0.81	−0.01	0.12	−1.24	−0.01	0.12	−1.03
alpha[6]	0.07	0.05	0.42	0.07	0.05	−0.15	0.07	0.05	0.36	0.07	0.05	0.97
alpha[7]	−0.31	0.18	−0.94	−0.31	0.19	−1.52	−0.3	0.19	−0.56	−0.31	0.19	−2.07
alpha[8]	−0.34	0.15	0.61	−0.34	0.15	−0.79	−0.33	0.15	−0.43	−0.34	0.16	−1.04
alpha[9]	−0.02	0.14	−0.59	−0.02	0.14	0.53	−0.02	0.14	−1.3	−0.02	0.14	−1.54
alpha[10]	0.18	0.07	−0.5	0.18	0.07	0.11	0.18	0.07	−0.84	0.18	0.07	0.02
alpha[11]	0.36	0.13	−1.86	0.36	0.13	−0.55	0.35	0.13	−0.55	0.36	0.13	−0.22
alpha[12]	−0.01	0.08	1.29	−0.01	0.08	−0.33	−0.01	0.08	2.5	−0.01	0.08	3.02
alpha[13]	−0.08	0.06	0.11	−0.09	0.06	−0.57	−0.09	0.06	2.07	−0.09	0.06	1.51
sd.p.site	0.96	0.12	2.34	0.95	0.12	0.12	0.95	0.13	1.61	0.95	0.12	−2.33
sd.p.survey	0.31	0.07	−1.22	0.31	0.07	−0.19	0.32	0.08	−1.36	0.32	0.07	−0.76

	block_AFSS				block_RW			jags_like_nimble			jags			nimble		
	Mean		*SD*	*Z*	Mean	*SD*	*Z*	Mean	*SD*	*Z*	Mean	*SD*	*Z*	Mean	*SD*	*Z*
Latent states sampled																
phi		0.97	0.02	0.03	0.97	0.02	−1.21	0.97	0.02	3.66	0.97	0.02	−1.39	0.97	0.02	−2.83
beta0		3.18	0.26	1.36	3.23	0.27	1.2	3.2	0.27	0.1	3.29	0.29	−2.94	3.19	0.29	2.66
beta[1]		−0.38	0.24	1.86	−0.37	0.23	0.25	−0.4	0.23	−0.55	−0.33	0.25	−2.48	−0.38	0.23	2.31
beta[2]		−0.05	0.19	0.96	−0.06	0.18	−1.79	−0.08	0.17	−0.39	−0.05	0.19	−1.65	−0.05	0.17	0.36
beta[3]		0.11	0.11	0.21	0.12	0.11	−1.04	0.12	0.11	−0.24	0.12	0.11	−2.05	0.11	0.11	2.04
beta[4]		−0.26	0.09	−0.98	−0.27	0.09	0.88	−0.26	0.09	0.15	−0.27	0.09	1.92	−0.26	0.09	−2.74
beta[5]		−0.89	0.73	0.04	−0.83	0.73	−0.94	−0.87	0.73	1.33	−0.83	0.73	−0.55	−0.85	0.75	−0.21
beta[6]		−0.06	0.11	−0.03	−0.05	0.11	−0.37	−0.05	0.11	−0.26	−0.05	0.11	−2.09	−0.05	0.11	3.08
beta[7]		−0.16	0.1	−0.7	−0.16	0.1	−0.75	−0.16	0.1	−0.25	−0.16	0.1	2.24	−0.15	0.1	−3.16
sd.lam		0.4	0.08	−0.54	0.38	0.09	−0.32	0.4	0.08	−1.2	0.37	0.1	6.56	0.4	0.08	−3.92
mean.p[1]		0.31	0.07	−0.52	0.29	0.07	−0.64	0.3	0.07	−1.16	0.27	0.07	3.45	0.3	0.08	−3.48
mean.p[2]		0.23	0.05	−0.56	0.22	0.06	−0.72	0.23	0.06	−1.16	0.21	0.06	3.31	0.23	0.06	−3.56
mean.p[3]		0.18	0.04	−0.59	0.17	0.05	−0.87	0.18	0.05	−1.08	0.16	0.05	3.21	0.18	0.05	−3.58
alpha[1]		−1.54	0.34	−1.64	−1.53	0.32	0.18	−1.51	0.33	0.61	−1.55	0.34	2.55	−1.55	0.32	−0.83
alpha[2]		−0.54	0.29	−0.39	−0.51	0.29	2.13	−0.49	0.29	1.08	−0.51	0.3	1.9	−0.55	0.28	0.35
alpha[3]		−0.1	0.13	1.49	−0.1	0.13	2.37	−0.1	0.13	−1.01	−0.1	0.13	−0.68	−0.1	0.13	−0.5
alpha[4]		0.07	0.07	−0.14	0.07	0.07	0.93	0.07	0.07	−0.11	0.07	0.07	3.32	0.07	0.07	1.08
alpha[5]		−0.01	0.12	−1.71	−0.01	0.12	0.37	−0.01	0.12	−0.28	−0.01	0.12	0.99	0	0.12	−0.62
alpha[6]		0.07	0.05	−0.22	0.07	0.05	−0.5	0.07	0.05	−0.53	0.07	0.05	−1.87	0.07	0.05	0.34
alpha[7]		−0.32	0.19	0.89	−0.32	0.19	−0.58	−0.31	0.19	−0.91	−0.31	0.18	−3.29	−0.3	0.19	−0.8
alpha[8]		−0.34	0.16	−0.78	−0.34	0.16	−3.45	−0.35	0.16	0.18	−0.34	0.15	−3	−0.33	0.16	1.34
alpha[9]		−0.02	0.14	−0.75	−0.02	0.14	−0.68	−0.02	0.14	−0.53	−0.02	0.14	−0.02	−0.02	0.14	−0.05
alpha[10]		0.19	0.07	−0.21	0.19	0.07	0.08	0.19	0.07	−0.9	0.18	0.07	−0.39	0.19	0.07	−0.65
alpha[11]		0.36	0.13	0.49	0.36	0.13	1.08	0.36	0.13	−0.8	0.36	0.13	0.2	0.35	0.13	−0.26
alpha[12]		−0.02	0.08	−1.72	−0.01	0.08	0.53	−0.01	0.08	1.38	−0.01	0.08	−0.36	−0.01	0.08	2.12
alpha[13]		−0.09	0.06	0.57	−0.09	0.06	0.79	−0.09	0.06	1.8	−0.08	0.06	−0.95	−0.09	0.06	0.62
sd.p.site		0.95	0.12	0.35	0.96	0.12	1.4	0.95	0.12	2.72	0.95	0.12	−2.87	0.95	0.12	−1.25
sd.p.survey		0.34	0.07	−0.27	0.33	0.07	−0.08	0.34	0.07	−2.06	0.31	0.07	3.96	0.34	0.08	−4.36

**Table A8:** Zero‐inflated N‐mixture model including species‐specific parameters and excluding hyperparameters (less hierarchical) posterior mean, *SD*, and Geweke *Z*‐score for chain convergence

	block_AFSS			block_RW			jags_like_nimble			nimble		
	Mean	*SD*	*Z*	Mean	*SD*	*Z*	Mean	*SD*	*Z*	Mean	*SD*	*Z*
Latent state integration												
phi	0.92	0.02	0.67	0.92	0.02	−0.74	0.92	0.02	−1.49	0.92	0.02	−0.04
beta0	2.71	0.11	0.6	2.71	0.11	0.7	2.71	0.11	0.04	2.71	0.11	−1.68
beta[1]	−0.97	0.12	−0.62	−0.97	0.12	1.54	−0.97	0.12	2.04	−0.97	0.12	0.07
beta[2]	−0.17	0.11	−1.71	−0.17	0.11	0.06	−0.17	0.11	1.77	−0.17	0.11	1.42
beta[3]	0.1	0.06	0.76	0.1	0.06	−1.49	0.1	0.06	0.71	0.1	0.06	1.13
beta[4]	−0.16	0.05	0.81	−0.16	0.05	2.13	−0.16	0.05	−0.63	−0.16	0.05	−0.49
beta[5]	−1.11	0.42	−0.83	−1.1	0.42	−0.15	−1.12	0.42	−0.03	−1.12	0.42	0.5
beta[6]	−0.02	0.06	0.27	−0.02	0.06	−1.66	−0.02	0.06	0.78	−0.02	0.06	1.06
beta[7]	−0.07	0.05	0.27	−0.07	0.05	2.13	−0.07	0.05	−0.81	−0.07	0.05	−0.28
mean.p[1]	0.59	0.03	−0.56	0.59	0.03	−0.46	0.59	0.03	0	0.59	0.03	2.73
mean.p[2]	0.5	0.03	−0.72	0.5	0.03	−0.58	0.5	0.03	−0.11	0.5	0.03	2.8
mean.p[3]	0.42	0.03	−0.63	0.42	0.03	−0.78	0.42	0.03	0.12	0.42	0.03	2.7
alpha[1]	−0.35	0.19	0.12	−0.35	0.19	−1.62	−0.35	0.19	−1.77	−0.35	0.19	−0.16
alpha[2]	−0.4	0.18	1.39	−0.41	0.18	0.39	−0.4	0.18	−1.75	−0.4	0.18	−1.52
alpha[3]	−0.26	0.1	−0.29	−0.26	0.1	0.79	−0.26	0.1	−0.6	−0.26	0.1	0.54
alpha[4]	0.12	0.06	0.4	0.12	0.06	1.52	0.12	0.06	−1.8	0.12	0.06	−0.63
alpha[5]	0.12	0.07	−1.07	0.12	0.07	0.43	0.12	0.07	0.91	0.12	0.07	−0.26
alpha[6]	0.04	0.03	0.2	0.04	0.03	1.29	0.03	0.03	0.58	0.04	0.03	−2.87
alpha[7]	−0.55	0.15	−0.87	−0.55	0.14	−1.58	−0.55	0.15	2.28	−0.55	0.14	0.32
alpha[8]	−0.34	0.11	−0.64	−0.34	0.11	−1.41	−0.34	0.11	2.28	−0.34	0.11	0
alpha[9]	0.07	0.09	1.28	0.07	0.09	−0.3	0.07	0.09	0.58	0.07	0.1	2.01
alpha[10]	0.15	0.04	−0.47	0.15	0.04	1.15	0.15	0.04	0.39	0.15	0.04	−2.27
alpha[11]	0.3	0.08	1.84	0.3	0.08	−0.58	0.3	0.08	−0.42	0.3	0.08	2.33
alpha[12]	−0.05	0.06	−0.33	−0.05	0.06	−0.6	−0.05	0.06	−1.28	−0.05	0.06	0.25
alpha[13]	−0.1	0.04	−0.92	−0.1	0.04	−0.69	−0.1	0.04	0.8	−0.1	0.04	1

	block_AFSS			block_RW			jags_like_nimble			jags			nimble		
	Mean	*SD*	*Z*	Mean	*SD*	*Z*	Mean	*SD*	*Z*	Mean	*SD*	*Z*	Mean	*SD*	*Z*
Latent states sampled															
phi	0.92	0.02	0.21	0.92	0.02	−1.29	0.92	0.02	−2.02	0.92	0.02	−1.91	0.92	0.02	0.56
beta0	2.71	0.11	1.08	2.71	0.11	0.47	2.71	0.11	0.2	2.71	0.11	−0.23	2.71	0.11	−1.41
beta[1]	−0.98	0.12	0.13	−0.97	0.12	0.24	−0.97	0.12	0.55	−0.97	0.12	1.3	−0.98	0.12	0.91
beta[2]	−0.18	0.11	−0.29	−0.18	0.11	−0.35	−0.18	0.11	0.42	−0.17	0.11	1.47	−0.18	0.11	1.33
beta[3]	0.1	0.06	−0.7	0.1	0.06	−0.41	0.1	0.06	0.1	0.1	0.06	0.46	0.1	0.06	1.57
beta[4]	−0.16	0.05	−0.11	−0.16	0.05	2.01	−0.16	0.05	−0.31	−0.16	0.05	−0.74	−0.16	0.05	−1.25
beta[5]	−1.12	0.42	1.28	−1.12	0.42	−0.52	−1.11	0.42	0.54	−1.11	0.42	0.45	−1.11	0.42	1.72
beta[6]	−0.01	0.06	−1.05	−0.01	0.06	−0.87	−0.01	0.06	−0.02	−0.01	0.06	0.28	−0.02	0.06	1.58
beta[7]	−0.07	0.05	−0.6	−0.07	0.05	2.29	−0.07	0.05	−0.08	−0.07	0.05	−0.66	−0.07	0.05	−1.2
mean.p[1]	0.6	0.03	−1.33	0.6	0.03	−0.61	0.6	0.03	−0.95	0.6	0.03	0.19	0.6	0.03	0.74
mean.p[2]	0.5	0.03	−1.09	0.5	0.03	−0.72	0.5	0.03	−0.95	0.5	0.03	0.33	0.5	0.03	0.61
mean.p[3]	0.42	0.03	−1.15	0.42	0.03	−0.67	0.42	0.03	−0.89	0.42	0.03	0.28	0.42	0.03	0.74
alpha[1]	−0.34	0.19	0.31	−0.35	0.19	−0.79	−0.34	0.19	−0.28	−0.35	0.19	−0.47	−0.34	0.19	−1.84
alpha[2]	−0.39	0.18	0.9	−0.4	0.18	0.38	−0.39	0.19	0.1	−0.4	0.18	−0.73	−0.39	0.19	−1.99
alpha[3]	−0.26	0.1	−1.18	−0.26	0.1	−0.77	−0.26	0.1	0.07	−0.26	0.1	−0.47	−0.27	0.1	1.56
alpha[4]	0.12	0.06	−0.33	0.12	0.06	−0.77	0.12	0.06	1.77	0.12	0.06	0.18	0.12	0.06	−2.18
alpha[5]	0.12	0.07	−0.41	0.12	0.07	0.49	0.12	0.07	−0.23	0.12	0.07	0.09	0.12	0.07	0.21
alpha[6]	0.03	0.03	1.27	0.03	0.03	−0.27	0.03	0.03	0.51	0.03	0.03	0.01	0.03	0.03	0.48
alpha[7]	−0.55	0.15	−0.58	−0.55	0.14	1.08	−0.55	0.14	−0.97	−0.55	0.14	−0.74	−0.55	0.14	2.41
alpha[8]	−0.34	0.11	−0.15	−0.34	0.11	0.43	−0.34	0.11	−0.33	−0.34	0.11	−0.34	−0.34	0.11	1.29
alpha[9]	0.07	0.09	−1.07	0.07	0.1	0.39	0.07	0.1	1.54	0.07	0.09	1.3	0.07	0.09	1.16
alpha[10]	0.15	0.04	0.16	0.15	0.04	−0.72	0.15	0.04	0.89	0.15	0.04	−0.14	0.15	0.04	0.56
alpha[11]	0.3	0.08	−1.27	0.3	0.08	−0.59	0.3	0.08	1.24	0.3	0.08	1.08	0.3	0.08	0.64
alpha[12]	−0.05	0.06	0.55	−0.05	0.06	−0.48	−0.05	0.06	−1.75	−0.05	0.06	0.17	−0.05	0.06	−1.11
alpha[13]	−0.1	0.04	1.36	−0.1	0.04	1.11	−0.1	0.04	−2.15	−0.1	0.04	0.04	−0.1	0.04	0.43
